# Protein–Ligand Interactions in Cardiometabolic Drug Targets: Focus on Weight Loss and Cardioprotection

**DOI:** 10.3390/molecules30214240

**Published:** 2025-10-30

**Authors:** Errikos Petsas, Despoina P. Kiouri, Nikitas Georgiou, Gerasimos Siasos, Thomas Mavromoustakos, Christos T. Chasapis

**Affiliations:** 1 Laboratory of Organic Chemistry, Department of Chemistry, National and Kapodistrian University of Athens, 11571 Athens, Greece; errpets@chem.uoa.gr (E.P.); despoina.kiouri.99@gmail.com (D.P.K.); nikitasgalleti93@hotmail.com (N.G.); tmavrom@chem.uoa.gr (T.M.); 2 Center for Interdisciplinary Biosciences, Technology and Innovation Park, P.J. Safarik University in Kosice, 040 01 Kosice, Slovakia; 3 3rd Department of Cardiology, Thoracic Diseases General Hospital Sotiria, Medical School, National and Kapodistrian University of Athens, 11527 Athens, Greece; gsiasos@med.uoa.gr

**Keywords:** cardiometabolic diseases, multi-target drug design, GLP-1 receptor, GIP receptor, FGFR1/β-Klotho, PCSK9 inhibition, NF-κB pathway, NLRP3 inflammasome

## Abstract

Cardiometabolic diseases (CVDs) are the leading cause of premature mortality and disability worldwide, arising from of cardiovascular and metabolic dysregulation. This review focuses on six critical therapeutic targets established in cardiometabolic regulation: GLP-1R, GIPR, FGFR1/β-Klotho, PCSK9, NF-κB, and the NLRP3 inflammasome. Drawing on curated structural datasets, we analyze the mechanisms of action and map key binding domain features that govern ligand efficacy and specificity. Dual GLP-1R/GIPR agonists, such as tirzepatide, demonstrate superior outcomes in glycemic control and weight reduction. Concurrently, inhibiting PCSK9, NF-κB, and NLRP3 helps to lower cholesterol and reduce harmful inflammation, offering cardioprotection. Structural analysis across these targets reveals complementary motifs (aromatic, hydrophobic, and polar residues). These insights guide the rational design of next-generation multi-target ligands (molecules capable of modulating two or more biological targets involved in related disease pathways, producing integrated therapeutic effects). Such integrated agents are promising for providing combined cardiovascular and metabolic benefits, thus reducing the risks associated with complex therapeutic drug combinations.

## 1. Introduction

Cardiovascular diseases (CVDs) remain the leading cause of premature mortality and disability worldwide, with a steadily increasing prevalence and a profound socioeconomic impact [1]. Cardiovascular, or heart diseases, influence the heart and the blood vessels and remain asymptomatic for long periods of time [2]. This umbrella term contains a variety of heterogeneous pathologies including coronary artery disease (CAD), cerebrovascular disease, peripheral artery disease (PAD), and aortic atherosclerosis [2]. Cardiovascular pathologies are closely related to metabolic diseases and both can be caused by hereditary and lifestyle factors, thus the establishment of the term “cardiometabolic diseases”, the intricate interplay between the dysregulation of the metabolic and cardiovascular system [3]. These different health problems share many of the same risk factors, such as insulin resistance, hypertension, obesity, and dyslipidemia that result in overlapping damage to their respective pathophysiological pathways [4]. Despite advances in monotherapies for glycemic control and lipid lowering, residual cardiovascular risk and limited weight reduction efficacy underscore the need for novel pharmacological strategies a shift toward integrated therapeutic approaches.

Numerous studies have shown that certain antihyperglycemic drugs can aid in weight loss in people with type 2 diabetes, including biguanides, alpha-glucosidase inhibitors, DPP-4 inhibitors, SGLT-2 inhibitors, and GLP-1 receptor agonists [5]. Based on their effect on weight loss, the diabetes drugs are classified into three groups: Mild (metformin, acarbose, empagliflozin and exenatide), Moderate (canagliflozin, ertugliflozin, dapagliflozin and dulaglutide) and strong weight loss effect (liraglutide, semaglutide and tirzepatide) [6]. Recent studies have also demonstrated that the use of anti-obesity medications effectively alleviates the high prevalence of cardiac disease [7,8]. Guo et al. [9] performed an extensive meta-analysis which revealed that every 5 kg weight loss that resulted from anti-obesity treatment reduced the risk of myocardial infarction, stroke and heart failure compared to the placebo. A few years ago, empagliflozin (i.e., a diabetes drug) was also approved for prescription in heart failure cases, even in cases when the patient is not diabetic [10].

Another popular strategy for the management of diabetes and metabolic conditions is combination therapy that involves the co-administration of two or more drugs to enhance the effectiveness of a single drug [11]. However, unexpected problems can occasionally occur because of the intricacy of the combination therapy products, such as antagonism, drug–drug interactions and thus augmentation of side effects [12]. The latter highlights the need for novel drugs that can be used for the treatment of closely related condition without the unwanted effects that accompany therapeutic drug combinations. scenarios. Advances in structural biology, computational docking, and ligand profiling have enabled the rational design of more selective and potent molecules. Understanding these interactions at the molecular level is therefore critical for optimizing therapeutic outcomes and minimizing adverse effects.

In this context, the present review focuses on six therapeutic protein targets with established roles in cardiometabolic regulation: Glucagon-like peptide-1 receptor (GLP-1R), Glucose-dependent insulinotropic polypeptide receptor (GIPR), Fibroblast Growth Factor Receptor 1-Klotho (FGFR1/β-Klotho), Proprotein convertase subtilisin/kexin type 9 (PCSK9), Nuclear factor NF-kappa-B p65 subunit (NF-κB), and the NACHT, LRR and PYD domains-containing protein 3 (NLRP3) inflammasome. Drawing on curated structural datasets, we classify known ligands, analyze their mechanisms of action, and map the key binding domain features that govern efficacy and specificity. Emerging paradigms in multi-target ligand design are also explored, with particular attention to dual-acting molecules capable of engaging multiple nodes within cardiometabolic networks. These agents are considered promising for providing integrated cardiovascular and metabolic benefits while reducing the risks and complexity associated with polypharmacy.

Although previous cardiometabolic reviews have examined incretin biology, lipid regulation, or inflammation separately, an integrated structural perspective across multiple targets remains lacking. This review bridges these domains by systematically comparing ligand–protein complexes of six therapeutically validated targets—GLP-1R, GIPR, FGFR1/β-Klotho, PCSK9, NF-κB, and NLRP3—to highlight convergent binding features and pharmacophoric motifs. Our aim is to delineate how multi-target ligand design can simultaneously achieve glycemic control, weight reduction, and cardioprotection, offering a framework for next-generation cardiometabolic therapeutics.

## 2. Therapeutic Targets and Mechanistic Insights

### 2.1. Glucagon-like Peptide-1 Receptor (GLP-1R)

The glucagon-like peptide-1 receptor (GLP-1R), a class B1 G protein-coupled receptor (GPCR), exhibits a conserved two-domain architecture crucial for ligand binding and activation. The extracellular domain (ECD) mediates initial ligand recognition, whereas the transmembrane domain (TMD) orchestrates conformational changes that enable G protein coupling and downstream signaling. Functionally, GLP-1R activation by its endogenous agonist GLP-1 promotes insulin secretion, suppresses glucagon release, slows gastric emptying, and induces satiety, thereby making it a pivotal therapeutic target in type 2 diabetes mellitus (T2DM) and obesity. Beyond metabolic regulation, GLP-1R signaling has been associated with neurotrophic effects, anti-inflammatory actions, and renal benefits. Clinically, GLP-1R agonists (GLP-1RAs cardiovascular protection) such as exenatide, liraglutide, dulaglutide, and semaglutide have demonstrated strong efficacy in lowering HbA1c and inducing weight loss, while next-generation multi-target agonists like tirzepatide (GLP-1R/GIPR dual agonist) and retatrutide (GLP-1R/GIPR/GCGR triagonist) provide superior glycemic and weight-control outcomes. In parallel, small-molecule GLP-1R modulators are under investigation: negative allosteric modulators suppress receptor activation by restricting critical conformational changes, whereas positive allosteric modulators enhance partial agonist efficacy, offering new strategies for precision therapy. More broadly, GLP-1R, together with the glucose-dependent insulinotropic polypeptide receptor (GIPR) and the glucagon receptor (GCGR), forms a central endocrine network regulating energy balance and insulin sensitivity, and their selective or combined modulation underpins modern pharmacological interventions against metabolic disorders [13].

GLP-1R agonists represent one of the most clinically validated classes within the cardiometabolic field. Their success derives from a favorable balance between efficacy and safety, as they achieve robust glycemic control, appetite suppression, and cardiovascular benefit with a well-characterized side-effect profile. Structural stabilization of the active G-protein-biased receptor conformation and resistance to DPP-IV degradation have been key determinants of their therapeutic performance. The predictable pharmacokinetics and receptor recycling dynamics of peptide agonists such as semaglutide and tirzepatide further support sustained metabolic improvements with minimal risk of desensitization. These features collectively explain the superior clinical outcomes of GLP-1R agonists compared with other incretin-based or peptide-derived therapeutics.

### 2.2. Glucose-Dependent Insulinotropic Polypeptide Receptor (GIPR)

The glucose-dependent insulinotropic polypeptide receptor (GIPR) is a class B G protein-coupled receptor that mediates the actions of GIP, one of the two primary incretin hormones. It is expressed in pancreatic islets, adipose tissue, and the central nervous system, exerting diverse roles in glucose homeostasis and energy balance. In pancreatic β-cells, GIPR activation enhances glucose-stimulated insulin secretion, while in α- and δ-cells it regulates glucagon and somatostatin release, respectively. In adipose tissue, GIPR signaling influences lipid metabolism, though evidence from genetic and pharmacological models remains conflicting, as both GIPR agonism and antagonism have been shown to reduce body weight. Similarly, the glucagon-like peptide-1 receptor (GLP-1R) is well established as a therapeutic target for type 2 diabetes and obesity, with GLP-1R agonists effectively lowering glucose levels and promoting weight loss. The pharmacological landscape now also includes GIPR antagonists, which may act by enhancing GLP-1R activity, and GIPR agonists that can desensitize receptor signaling in a manner resembling antagonism. More recently, dual GLP-1R/GIPR agonists, specifically tirzepatide, have demonstrated synergistic effects on glycemic control and weight reduction by engaging both incretin pathways simultaneously, thereby offering superior metabolic benefits compared to single receptor agonists [14].

In contrast to GLP-1R, the translational progress of GIPR agonists has been slower and more complex. Endogenous GIP shows context-dependent effects, stimulating insulin secretion in euglycemia but potentially promoting adipogenesis under chronic hyperinsulinemia. However, the development of dual GLP-1R/GIPR agonists such as tirzepatide has revived interest in this pathway, as structural studies revealed complementary receptor engagement leading to enhanced metabolic efficiency and reduced gastrointestinal side effects. The improved bias toward G-protein signaling and synergistic activation with GLP-1R provide a mechanistic explanation for the superior efficacy of dual incretin agonists compared with single-target molecules.

### 2.3. Fibroblast Growth Factor Receptor 1/β-Klotho Complex (FGFR1/β-Klotho)

Fibroblast Growth Factor Receptor 1 (FGFR1) is a prototypical member of the receptor tyrosine kinase family that governs diverse cellular processes, including proliferation, differentiation, survival, and metabolism. Structurally, FGFR1 consists of a conserved extracellular region that features three immunoglobulin-like (Ig) domains (D1–D3), a single transmembrane helix, and an intracellular tyrosine kinase domain responsible for its catalytic activity. Upon ligand binding and receptor dimerization, FGFR1 activates multiple downstream pathways such as MAPK/ERK, PI3K–AKT, PLCγ, and STAT, thereby integrating mitogenic and metabolic signals. Τhe formation of the FGFR1/β-Klotho complex mimics the metabolic hormone FGF21 and plays a critical role in lipid metabolism through structural interactions involving the Ig-like domain pocket. Dysregulation of FGFR1 signaling has been linked to oncogenesis, developmental anomalies, and metabolic disorders, positioning FGFR1 as both a biomarker and a key therapeutic target. The receptor can be targeted pharmacologically through opposing strategies: Agonists, including natural ligands such as FGF1, FGF2, and FGF21, have been shown to activate FGFR1 and improve glucose and lipid homeostasis in models of obesity and type 2 diabetes. Conversely, selective FGFR inhibitors, such as erdafitinib, have entered clinical practice to suppress aberrant FGFR1 activity in cancers characterized by receptor overexpression, mutation, or gene amplification. Taken together, FGFR1 functions as a central regulator of both metabolic and proliferative signaling, and its pharmacological modulation by both agonists and antagonists highlights its broad translational potential in oncology and metabolic disease therapy [15].

FGFR1/β-Klotho signaling offers potent metabolic regulation through modulation of glucose uptake, lipid oxidation, and thermogenesis; however, clinical translation has been hampered by narrow therapeutic windows and off-target mitogenic effects. The receptor’s pleiotropic downstream signaling (MAPK, STAT, and PI3K pathways) complicates selectivity, as subtle structural modifications can shift the balance between beneficial and proliferative responses. Partial agonists and engineered FGF21 analogs have been developed to mitigate these risks, but long-term safety data remain limited. Compared with incretin-based drugs, FGFR1 agonists currently face greater translational barriers due to receptor complexity and potential tissue cross-activation.

### 2.4. Proprotein Convertase Subtilisin/Kexin Type 9 (PCSK9)

Proprotein convertase subtilisin/kexin type 9 (PCSK9), the ninth member of the proprotein convertase (PC) family, plays a pivotal role in cholesterol regulation and cardiovascular health [16]. Members of this protease family function by converting inactive secretory precursors into biologically active products, including neuropeptides, prohormones, cytokines, growth factors, cell-surface proteins, and serum proteins [16]. Structural characterization of PCSK9 has revealed three distinct domains: the prodomain (amino acids 31–152), the catalytic domain (amino acids 153–421), and the C-terminal Cys/His-rich domain (CHRD; amino acids 453–692) [17]. Although PCSK9 promotes degradation of the low-density lipoprotein receptor (LDLR), its catalytic activity is not essential for this process [18]. Predominantly expressed in the liver, intestine, and kidney, PCSK9 was first recognized as a key regulator of lipid metabolism when gain-of-function mutations in its gene were linked to familial hypercholesterolemia and an increased risk of coronary artery disease [18]. Since its discovery, PCSK9 has shifted from being considered a rare cause of hypercholesterolemia to becoming one of the most effective therapeutic targets for cholesterol reduction [19]. Clinical outcome trials have confirmed that PCSK9 inhibition significantly lowers circulating LDL levels and reduces atherosclerotic cardiovascular disease (ASCVD) risk [19]. By preventing the interaction between PCSK9 and LDLR, PCSK9 inhibitors block receptor degradation, thereby enhancing LDLR availability on hepatocytes and promoting the clearance of plasma LDL cholesterol [20].

PCSK9 inhibition exemplifies structure-based success in lipid-lowering therapy. The elucidation of its crystal structure enabled rational antibody design (alirocumab, evolocumab) that precisely blocks the PCSK9–LDLR interface. Clinically, these agents achieve substantial LDL-cholesterol reduction and cardiovascular risk lowering, often surpassing statins in high-risk populations. Nevertheless, the peptide nature of antibodies limits oral bioavailability, and small-molecule inhibitors face challenges in reproducing the high-affinity binding achieved by biologics. Compared with incretin or FGF-based therapies, PCSK9 targeting demonstrates exceptional efficacy in lipid modulation but limited metabolic pleiotropy.

### 2.5. Nuclear Factor NF-Kappa-B p65 Subunit (NF-κB)

Nuclear factor-kappa B (NF-κB) represents a critical family of five ubiquitous transcription factors central to regulating gene expression in mammalian cell types. All members are characterized by the presence of the Rel homology domain, which is notably found in the p65 subunit (RelA). These factors can form various homo- or heterodimers. The classical NF-κB complex, comprising the p65/p50 heterodimer, is primarily responsible for binding to DNA and orchestrating the transcription of genes critical for immune responses, cellular proliferation, and development [21]. The transcriptional potency of the NF-κB complex stems primarily from the p65 subunit’s carboxy-terminal activation domain. This domain facilitates the recruitment of coactivators necessary for interactions with the core transcriptional machinery. NF-κB activation is triggered by a wide range of cellular stimuli relevant to cardiometabolic disease, including cytokines, reactive oxygen species (ROS), and oxidized low-density lipoprotein (ox-LDL). Under resting conditions, NF-κB is sequestered in the cytoplasm by inhibitory proteins, such as IκBα. Upon stimulation, IκBα is degraded, allowing the free p65/p50 heterodimer to translocate into the nucleus, initiating the expression of pro-inflammatory genes. Due to its role as a key regulator in inflammation and cardiovascular remodeling, NF-κB dysregulation is implicated in numerous pathological processes. Therefore, pharmacological inhibition of NF-κB—specifically targeting the DNA-binding ability of the p65/p50 heterodimer—serves as a robust therapeutic strategy to suppress the transcription of pro-inflammatory cytokines and attenuate downstream inflammatory signaling crucial in cardiometabolic pathologies [22].

NF-κB remains a central inflammatory node linking obesity, insulin resistance, and atherogenesis. Pharmacological inhibition through natural or synthetic ligands (e.g., parthenolide derivatives) offers anti-inflammatory and metabolic benefits but also carries risks of immunosuppression due to NF-κB’s ubiquitous role in host defense. Structurally, most ligands bind covalently or allosterically within the p65 or IKK subunits, but achieving selectivity without affecting canonical immune functions remains challenging. Thus, while NF-κB modulation provides mechanistic insight into inflammation control, its translation into safe chronic therapies lags behind metabolic-targeted approaches such as GLP-1R or PCSK9 inhibition.

### 2.6. NACHT, LRR and PYD Domain-Containing Protein 3 (NLRP3) Inflammasome

The NLR family pyrin domain containing 3 (NLRP3) inflammasome is a multimeric cytosolic protein complex that plays a pivotal role in innate immune signaling [23]. It is composed of an amino-terminal pyrin (PYD) domain, a central nucleotide-binding and oligomerization domain (NACHT) with ATPase activity, and a carboxy-terminal leucine-rich repeat (LRR) domain [24]. Upon activation, the NLRP3 inflammasome promotes the recruitment and activation of caspase-1, resulting in the maturation and secretion of the proinflammatory cytokines interleukin-1β (IL-1β) and IL-18, as well as induction of pyroptosis, an inflammatory form of programmed cell death in sentinel immune cells [23]. A broad range of exogenous and endogenous signals have been shown to trigger NLRP3 activation, including microbial products such as pore-forming toxins and RNA, danger-associated molecules such as uric acid and ATP, and crystalline particles including silica, asbestos, and alum [25]. Although it represents the most extensively studied inflammasome pathway, the precise mechanisms underlying NLRP3 activation remain incompletely understood [25]. Over the decade, NLRP3 has been implicated in the pathogenesis of numerous disorders, including autoimmune diseases, central nervous system-related conditions, malignancies, and metabolic disorders [26]. Inhibitors targeting NLRP3 have demonstrated therapeutic potential by interfering with assembly of the NACHT domain, thereby suppressing IL-1β production under conditions of metabolic stress and attenuating maladaptive inflammation [27].

NLRP3 acts as a convergent sensor of metabolic stress, and its inhibition represents an emerging strategy for cardiometabolic inflammation. Small-molecule inhibitors targeting the NACHT domain have demonstrated effective blockade of inflammasome assembly and cytokine release, improving metabolic and cardiovascular parameters in preclinical models. However, the receptor’s structural plasticity and ATP-dependent activation make selectivity a persistent obstacle. Compared with upstream targets like GLP-1R or FGFR1, NLRP3 inhibition operates at the terminal stage of inflammatory signaling, offering strong anti-cytokine effects but with potential for immunological side reactions. Continued refinement of NLRP3-selective ligands is essential to balance efficacy with safety.

## 3. Classification of Known Ligands per Target

To provide a structured overview of the therapeutic peptides under investigation, this section presents a comparative summary of the key ligands, highlighting their principal pharmacological properties and distinctive molecular characteristics. The compounds included are Albiglutide, Avexitide, Dulaglutide, Exenatide, Glucagon, Liraglutide, Lixisenatide, Pramlintide, Semaglutide, and Tirzepatide, which collectively represent a broad spectrum of incretin mimetics and peptide-based agents with clinical relevance in metabolic disorders such as type 2 diabetes and obesity. By outlining their structural features, receptor selectivity, half-life, and clinical applications, Table 1 that follows aims to facilitate a direct comparison and to contextualize their therapeutic positioning within current pharmacological strategies.

**Table 1:** Bioavailability of the compounds, IC_50_ and chemical structures of them with Glucagon Like Peptide 1 Receptor.

Compound	Chemical Structures	IC_50_ (nM) with GLP1-R	Bioavailability Subcutaneous
Albiglutide	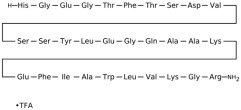	~0.61 nM [28]	30–50% [29]
Avexitide	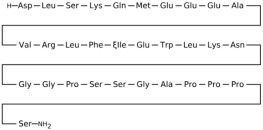	~1.9 nM [30]	Not reported
Dulaglutide	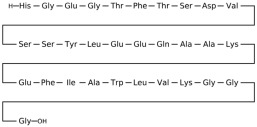	~18.2 nM [31]	65%
Exenatide	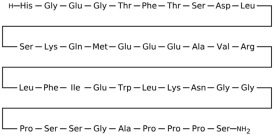	~3.22 nM [32]	65%
Glucagon	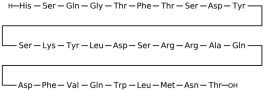	4.9 nM [33]	16% [34]
Liraglutide	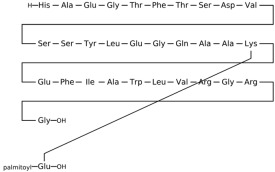	~0.5 nM [35]	55%
Lixisenatide	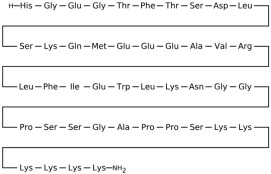	~1.4 nM [36]	2% [37]
Pramlintide	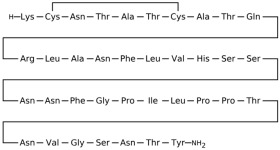	not reported	30–40%
Semaglutide	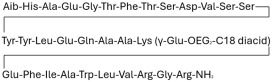	~0.15 nM [38]	89% [39]
Tirzepatide	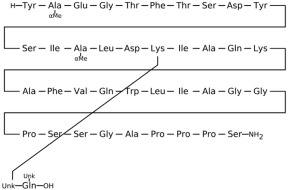	~0.26–3.3 nM [40]	81% [41]

### 3.1. Known Ligands of Glucagon-like Peptide-1 Receptor (GLP-1R)

#### 3.1.1. Dulaglutide

Dulaglutide, a long-acting GLP-1R agonist, structurally mimics endogenous GLP-1 and binds both domains cooperatively. Structural studies have shown that GLP-1R agonists like dulaglutide interact with the ECD via α-helical motifs and hydrophobic residues, stabilizing the ligand-receptor complex. This dual-domain engagement is a hallmark of peptide-based agonists and facilitates conformational rearrangement essential for downstream signaling [13,42].

Cryo-electron microscopy and X-ray crystallographic data reveal that dulaglutide binds to GLP-1R in a conformation similar to the native GLP-1 peptide, engaging both the N-terminal extracellular domain (ECD) and the upper regions of the transmembrane helices (TMD). The crystal structure of GLP-1R in complex with a GLP-1 analogue (PDB ID: 5VAI) demonstrates key ligand-receptor interactions that stabilize the active conformation. Critical residues involved in the binding pocket include Glu364, which forms salt bridges with positively charged ligand residues; Arg190 and Tyr152, which contribute to hydrogen bonding; and Trp297 in the extracellular loop 2 (W297^ECL2), which provides hydrophobic contact that anchors the ligand. Additional supportive interactions include Ser32 and His212 in the ECD, which help secure the N-terminal α-helix of dulaglutide, while Ile196 and Phe230 within the TMD contribute to hydrophobic core formation. These residues together form a deep orthosteric pocket, locking the ligand in a bioactive conformation and aligning the receptor for downstream Gs protein coupling. Moreover, the C-terminal extension of dulaglutide, a covalently linked Fc fragment, enhances serum stability and resists degradation by dipeptidyl peptidase-4 (DPP-4), contributing to its prolonged receptor occupancy and pharmacodynamic action [13].

The specific protein–ligand interactions between dulaglutide and GLP-1R translate directly into its pharmacological efficacy and clinical outcomes. By maintaining high-affinity binding and inducing prolonged activation of GLP-1R, dulaglutide effectively enhances insulin secretion, inhibits glucagon release, and delays gastric emptying—mechanisms critical for glycemic control in type 2 diabetes mellitus (T2DM) [43]. Furthermore, dulaglutide-mediated GLP-1R activation promotes neuroprotective and cardioprotective effects, which have been linked to downstream signaling pathways including cAMP/PKA, AMPK, and PI3K-Akt [44,45]. The sustained receptor engagement enabled by these structural interactions also underpins dulaglutide’s once-weekly dosing regimen and its durability in reducing β-cell stress without downregulating GLP-1R expression [42]. Thus, the structural compatibility between dulaglutide and GLP-1R ensures a therapeutic profile marked by efficacy, safety, and improved patient adherence.

#### 3.1.2. Exenatide

Exenatide (Ex-4), a synthetic analog of exendin-4, binds to GLP-1R in a two-domain interaction mode. The C-terminal region of exenatide primarily engages the extracellular domain (ECD) of GLP-1R for high-affinity binding, while the N-terminal portion inserts into the transmembrane (TM) domain to induce receptor activation [13,46] The receptor itself is structured with a characteristic seven-transmembrane helical bundle (7TM), connected by extracellular and intracellular loops that accommodate both peptide and small-molecule ligands. Structural studies show that Trp-cage motifs on exenatide enhance its stability and modulate the conformational flexibility required for binding, while the TM6 region of the receptor functions as the principal signal transduction hub [46].

**Figure 1:**
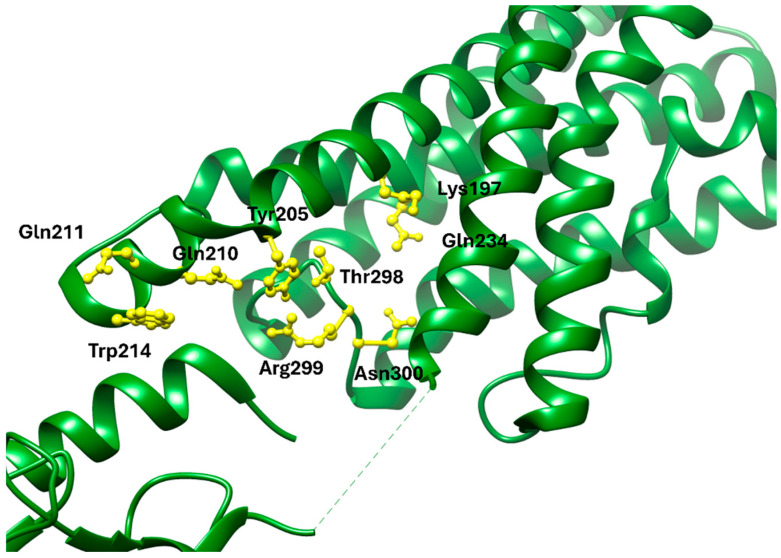
Cryo-EM structure of the human GLP-1 receptor (GLP1R) (PDB ID: 6X18). For clarity, only chain R (GLP1R) is shown, with all other deposited chains omitted. The receptor is rendered in ribbon representation (green) with exenatide. Amino acid residues defining the peptide-binding pocket (Lys197, Tyr205, Gln210, Gln211, Trp214, Gln234, Thr298, Arg299, and Asn300) are highlighted in ball-and-stick form (yellow), delineating the principal recognition interface for exenatide.

Cryo-electron microscopy (cryo-EM) and X-ray crystallography have provided detailed insights into the interaction between GLP-1R and exenatide. The structural configuration is well-documented in the Protein Data Bank under entries such as PDB: 6X18, which display the active-state GLP-1R bound to exenatide and related agonists [13,47]. These structures reveal that the ECD interacts with the α-helical region of exenatide, anchoring residues such as Tyr205, Glu364, and Trp33 (in the ECD), which contribute significantly to ligand recognition. Within the transmembrane bundle, residues Lys197, Tyr205, Gln210, Gln211, Trp214, Gln234, Thr298, Arg299, and Asn300 form a binding pocket that accommodates the N-terminal activation motif of the peptide (Figure 1) [46,47]. Moreover, the Trp25 residue in exenatide’s Trp-cage motif aligns structurally with the receptor’s extracellular loops, indicating a secondary stabilization role despite its minimal contribution to direct receptor engagement [46].

The structural interaction between exenatide and GLP-1R translates directly into therapeutic outcomes in metabolic and neurodegenerative diseases. By stabilizing GLP-1R in its active conformation, exenatide enhances glucose-stimulated insulin secretion, suppresses glucagon release, and slows gastric emptying—actions critical for managing type 2 diabetes mellitus [48,49]. The biased agonism of exenatide, favoring G protein signaling over β-arrestin recruitment, contributes to its beneficial effects on glucose metabolism while potentially reducing receptor desensitization and side effects such as nausea [47]. Furthermore, exenatide’s ability to cross the blood–brain barrier and engage GLP-1Rs in the CNS confers neuroprotective properties, including reductions in oxidative stress and inflammation, making it a candidate for addressing central nervous system disorders like Parkinson’s and Alzheimer’s diseases [49]. Thus, understanding the precise molecular interactions between GLP-1R and exenatide not only elucidates the basis for its clinical efficacy but also informs the design of next-generation agonists with improved therapeutic profiles.

#### 3.1.3. Glucagon

The GLP-1R adopts a conserved “secretin fold” in its ECD, stabilized by three disulfide bridges and key residues including Asp67, Trp72, and Pro86, which are essential for anchoring the C-terminal α-helix of GLP-1 [50]. Structural insights from cryo-EM and crystallography show that GLP-1 binds in a two-domain fashion: its C-terminal region interacts with the ECD while the N-terminal segment penetrates the transmembrane domain (TMD) to initiate receptor activation [51]. This bipartite engagement is similarly observed for dual GLP-1R/GCGR agonists such as MEDI0382 and SAR425899, which modify conserved motifs across both receptors to achieve dual activation [52].

High-resolution cryo-EM structures have revealed that both peptide (e.g., GLP-1, oxyntomodulin) and non-peptide agonists (e.g., PF 06882961) exploit overlapping receptor-binding surfaces, though their engagement strategies differ. Peptide agonists typically occupy the orthosteric site through extensive hydrophobic and hydrogen bonding interactions across ECL1, ECL2, and transmembrane helices 1 and 7 [53]. In contrast, non-peptide agonists like PF 06882961 use structured water networks and mimic the polar contacts of GLP-1, enabling them to trigger similar receptor conformational changes despite their smaller size. MEDI0382, a dual GLP-1R/GCGR agonist, engages the TM1-TM2 cleft with a lipid moiety that enhances its binding to GCGR, accounting for its higher potency and efficacy in metabolic regulation [52]. These distinct modes underscore the plasticity of class B1 GPCRs in accommodating diverse ligand chemotypes.

The structural nuances of GLP-1R and Glucagon ligand interactions have direct implications for therapeutic design. Biased agonism at GLP-1R, wherein ligands preferentially activate specific signaling pathways, can be tuned through targeted interactions at the extracellular surface and ECL regions, allowing for tailored pharmacodynamic profiles [51]. MEDI0382, by engaging both GLP-1R and GCGR, achieves superior metabolic effects—such as glycemic control and weight loss—by combining insulinotropic and thermogenic activities while mitigating glucagon-induced hyperglycemia [54,55]. These findings highlight the clinical promise of dual or multi-receptor agonists that leverage shared structural determinants while fine-tuning selectivity through engineered sequence variation [52]. Continued structural elucidation informs the rational design of next-generation metabolic therapeutics with improved efficacy and safety profiles.

#### 3.1.4. Liraglutide

Liraglutide, a long-acting GLP-1R agonist, binds to specific structural domains on the receptor to exert its therapeutic effects. Structural analyses revealed that the extracellular domain (ECD) of GLP-1R is critical for ligand recognition, with the C-terminal region of GLP-1 or its analogs, like liraglutide, binding deeply into a conserved hydrophobic pocket. When GLP-1 binds, it adopts an extended α-helical conformation from Thr13 to Val33, allowing precise interactions with receptor residues that differ slightly from those seen in antagonist-bound conformations [50,56].

Functionally, the binding mode of liraglutide to GLP-1R has been elucidated through structural studies that include crystallography data. The extracellular domain (ECD) of GLP-1R interacts directly with the C-terminal α-helical segment of liraglutide, a modification that enhances stability and resistance to enzymatic degradation. Specifically, the crystal structure of the GLP-1R ECD bound to GLP-1 (PDB ID: 3IOL) reveals that GLP-1 adopts a continuous α-helix from Thr13 to Val33, fitting into a hydrophobic groove formed by conserved residues on the receptor [50]. This interaction is critical for the initial high-affinity binding step, which is then followed by engagement of the ligand’s N-terminal domain with the receptor’s transmembrane core to trigger G-protein activation. The binding orientation of GLP-1 in 3IOL differs from that of Exendin-4, an agonist used in a prior structure (PDB ID: 3C5T), indicating subtle but important conformational distinctions that influence receptor activation and signaling efficiency [49].

From a therapeutic standpoint, the interaction between liraglutide and GLP-1R is crucial not only for its antidiabetic properties but also for its potential in treating conditions like nonalcoholic steatohepatitis (NASH) and obesity. For instance, liraglutide modulates hepatic inflammation and lipotoxicity via direct liver actions, independent of weight loss or improved glycemic control, likely through suppression of ceramide accumulation and alteration of gut microbiota [57]. These diverse outcomes are mediated by liraglutide’s capacity to act on central and peripheral GLP-1R populations, particularly in glutamatergic neurons, where it modulates appetite and body weight without compromising glycemic benefits [58,59].

#### 3.1.5. Albiglutide

Structural studies have revealed that GLP-1 and albiglutide bind in a two-domain model: the C-terminal portion of the ligand engages the ECD while the N-terminal portion interacts with the transmembrane domain, particularly transmembrane helices and extracellular loops [50,60]. Cryo-EM structures show GLP-1R activation involves conformational rearrangements including a kink in TM6 to facilitate G protein coupling, emphasizing the importance of conserved motifs such as the secretin fold and disulfide-stabilized helices in signal transduction [60,61].

Albiglutide, a long-acting GLP-1R agonist, is engineered as a dimer of GLP-1 (7–36) molecules fused to recombinant human albumin, which confers extended half-life and resistance to enzymatic degradation. Albiglutide exhibits altered binding kinetics due to steric effects and slower dissociation from the receptor, yet it retains sufficient agonist efficacy to activate downstream signaling cascades, including PKA-mediated insulin release from pancreatic β-cells [62,63]. This slow-binding characteristic allows for once-weekly dosing and reduces peak plasma fluctuations, mitigating gastrointestinal side effects and improving patient adherence [64].

The therapeutic outcome of albiglutide’s interaction with GLP-1R extends beyond glycemic control, reflecting the receptor’s pleiotropic signaling potential. By stimulating glucose-dependent insulin secretion, suppressing glucagon release, and delaying gastric emptying, albiglutide contributes to improved HbA1c levels and modest weight reduction [65]. Clinical data support its efficacy, with significant HbA1c reductions over placebo at both 30 mg and 50 mg weekly doses [62]. Moreover, the cardioprotective and β-cell-preserving effects observed in preclinical and clinical studies underscore GLP-1R’s central role as a therapeutic target in type 2 diabetes and its comorbidities [63,64].

#### 3.1.6. Lixisenatide

Lixisenatide, an exendin-4-based GLP1R agonist, engages key structural elements such as the extracellular loop 2 (ECL2), TM helices 1, 2, 6, and 7, and the ECD’s N-terminal region to trigger receptor activation [51,53]. Cryo-EM structures show that these peptide agonists are stabilized by a network of water-mediated hydrogen bonds that link the peptide’s N-terminus to the receptor core, ensuring specificity and efficacy. Importantly, distinct residues in the ECD modulate biased agonism, allowing lixisenatide to differentially recruit downstream effectors [51].

Lixisenatide binds to the GLP-1 receptor (GLP1R) using a mechanism analogous to GLP-1, engaging both the extracellular domain (ECD) and transmembrane (TM) regions of the receptor. Cryo-EM structures of GLP1R in active states, such as PDB IDs 6B3J, 6X18, and 6LN2, which show peptide agonists interacting with lixisenatide via N-terminal contacts and inserting into the orthosteric TM cavity to stabilize the active conformation [53]. Notably, although lixisenatide shares the first 37 amino acids with exendin-4, it includes a unique six-lysine C-terminal extension, which contributes to altered receptor engagement and trafficking properties. Pharmacologically, lixisenatide induces robust receptor internalization but slower recycling to the membrane compared to exendin-4, likely due to subtle structural differences in receptor clustering and endosomal sorting [53]. These variations in binding and trafficking may contribute to differences in sustained insulin secretion and biased signaling profiles among GLP-1 analogs.

Lixisenatide’s interactions with GLP1R not only underpin its glucose-lowering efficacy but also contribute to extrapancreatic effects such as neuroprotection and vascular modulation. It has demonstrated potent inhibition of postprandial glucagon and improved β-cell function while causing less gastrointestinal discomfort compared to longer-acting GLP1R agonists [66,67]. In neuronal tissues, lixisenatide exerts antioxidant and anti-inflammatory effects, partly via GLP1R-mediated upregulation of endothelial nitric oxide synthase (eNOS) and suppression of pro-apoptotic pathways, offering protection against ischemic injury [68,69].These findings underscore the therapeutic versatility of lixisenatide and its mechanistic reliance on GLP1R structural motifs to mediate both glycemic control and organ protection.

#### 3.1.7. Pramlintide

The glucagon-like peptide-1 receptor (GLP-1R) and amylin analog pramlintide engage distinct yet complementary structural and functional features that define their protein–ligand interactions and biological efficacy in metabolic disease therapy [70,71]. Pramlintide, an analog of the 37-amino acid hormone amylin, interacts predominantly with the calcitonin receptor (CALCR) complexed with receptor activity-modifying proteins (RAMPs) [72]. Structural features critical for pramlintide’s activity include its engineered proline residues that reduce fibrillogenesis while preserving key agonistic motifs for receptor binding in the area postrema and nucleus of the solitary tract [73]. These differences in receptor-binding architecture underscore the unique but convergent signaling pathways activated by these agents.

Molecular and physiological studies have revealed that both GLP-1R agonists and pramlintide modulate central appetite-regulatory circuits, though through distinct mechanisms and structural interactions. The GLP-1 receptor has been extensively characterized, with crystallographic data available for multiple ligand-bound states. Notably, the cryo-EM structure of human GLP-1R bound to GLP-1 and Gs protein (PDB ID: 6X18) reveals a key interaction at the extracellular domain and transmembrane helices, stabilizing the active conformation of the receptor and initiating intracellular signaling cascades. Additionally, structures such as 6LN2 and 5VAI provide insight into small-molecule and peptide ligand interactions within the GLP1R binding pocket. Conversely, pramlintide binds to amylin receptors formed by heterodimerization of the calcitonin receptor (CTR) with RAMPs (particularly RAMP1, 2, or 3). This structure illustrates how peptide agonists interact with the extracellular domain of the receptor, inducing activation. Together, these structural insights reveal that while GLP-1R agonists activate through well-defined GPCR conformational shifts, pramlintide engages class B GPCRs with RAMP-modulated binding, offering complementary and synergistic potential in combination therapies [70,71,74].

Pramlintide (an amylin analog), although less widely used due to its injection burden, has demonstrated efficacy in reducing caloric intake and improving control over eating in obese individuals [74]. Notably, combination therapies leveraging both GLP-1 and pramlintide have shown synergistic effects in enhancing weight loss [72,75].

#### 3.1.8. Semaglutide

Semaglutide, a long-acting GLP-1 receptor (GLP-1R) agonist, exhibits high binding affinity to GLP-1R through conserved structural motifs common to class B G protein-coupled receptors. Structural analyses using cryo-electron microscopy (cryo-EM) reveal that semaglutide adopts a helical conformation similar to endogenous GLP-1 and binds within the orthosteric site of GLP-1R, engaging both the extracellular domain (ECD) and the transmembrane (TM) domain. Notably, semaglutide’s synthetic modifications—such as an acylation at Lys26 with a C18 fatty diacid chain—contribute to its prolonged half-life and enhanced albumin binding [56,76]. These interactions involve key residues within TM1, TM2, and ECL2, stabilizing the active conformation of the receptor and facilitating Gs-protein coupling, as confirmed by structures at 2.5 Å resolution [76].

Semaglutide’s binding mode has been extensively studied using structural and computational tools, anchored by the X-ray structure of the GLP-1R-ECD in complex with GLP-1 (PDB: 4ZGM). These structural insights reveal that semaglutide engages GLP-1R through both its N-terminal α-helix and modified C-terminal extension, mimicking endogenous GLP-1 interactions while introducing stabilizing modifications. Key binding residues within the receptor include Tyr148 and Thr149 in transmembrane helix 1 (TM1); Arg190 and Glu138 in TM2; Tyr205 in extracellular loop 1 (ECL1); Arg299 in TM5; Glu364 and Arg380 in TM6 and ECL3 [77]. Modeling suggests the formation of a stable interfacial electrostatic scaffold, composed of four salt bridges at the GLP-1R-ECD interface, which is predicted to improve receptor activation. These findings underscore the importance of receptor-ligand dynamics and have guided the rational design of next-generation GLP-1R agonists [47,77].

The biological outcomes of GLP-1R and semaglutide binding are multifaceted, with therapeutic implications extending beyond glycemic control. In Alzheimer’s disease models, semaglutide binding to GLP-1R upregulates SIRT1 and GLUT4 expression in the hippocampus, enhancing glucose metabolism and reducing Aβ/tau pathology [78]. Similarly, in oncology, semaglutide reprograms tumor-associated macrophages via the GLP-1R/PPARG/ACSL1 pathway, promoting M1 polarization and suppressing tumor progression [79]. Clinically, semaglutide has been validated for its cardioprotective effects, reducing major adverse cardiovascular events in high-risk type 2 diabetes patients [80]. These therapeutic effects reflect the broad tissue expression of GLP-1R and highlight the clinical value of structurally informed ligand design [81].

#### 3.1.9. Avexitide

Avexitide (exendin 9–39) is a potent antagonist of the glucagon-like peptide-1 receptor (GLP-1R), a class B G protein-coupled receptor (GPCR) involved in the regulation of glucose homeostasis. GLP-1R features two key structural domains: an extracellular domain (ECD) responsible for initial ligand binding and a transmembrane domain (TMD) that mediates downstream signaling. Avexitide binds primarily to the orthosteric site within the ECD [82,83]. Structural and functional analyses of class B GPCRs support a two-step activation mechanism—avexitide disrupts the first step, namely high-affinity binding to the ECD, thus blocking downstream metabolic signaling [83].

Detailed binding studies have identified key residues in the GLP-1R that interact with avexitide. These include residues in the N-terminal region such as Trp33, Tyr69, and His89, which are essential for ligand recognition at the ECD, as well as Glu128, Arg190, and Tyr205 within the transmembrane helices that contribute to allosteric conformational locking in the inactive state [82,83]. By occupying the ECD without engaging the transmembrane core, avexitide effectively stabilizes an inactive receptor conformation and disrupts endogenous peptide binding. Moreover, functional assays have shown that avexitide prevents the ligand-induced outward movement of transmembrane helix 6 (TM6), acting as a competitive antagonist at the orthosteric site [83,84].

The clinical relevance of avexitide’s GLP-1R antagonism is underscored by its therapeutic use in treating postbariatric hypoglycemia (PBH), a condition characterized by excessive insulin secretion following gastric bypass surgery. By blocking GLP-1R, avexitide reduces postprandial insulin peaks and prevents hypoglycemia without significantly altering basal glucose levels [84]. In a randomized controlled trial, avexitide significantly raised glucose nadir and lowered insulin peaks during mixed-meal tolerance testing, leading to fewer symptomatic hypoglycemic episodes [84,85,86].

### 3.2. Fibroblast Growth Factor Receptor 1 (FGFR1)

This section provides a structured overview of the selected ligands, highlighting their fundamental properties and key pharmacological characteristics. The compounds under consideration include small molecule kinase inhibitors such as fostamatinib, erdafitinib, futibatinib, infigratinib, lenvatinib, nintedanib, pemigatinib, ponatinib, pralsetinib, regorafenib, and selpercatinib, as well as biologically active agents like heparin, palifermin, and romiplostim. These ligands represent diverse therapeutic classes, ranging from angiogenesis and fibroblast growth factor receptor (FGFR) inhibitors to receptor tyrosine kinase (RTK) modulators and hematopoietic growth factors. By summarizing their mechanisms of action, molecular targets, and clinical relevance, Table 2 facilitates a comparative analysis that underscores their distinct roles in targeted therapies and biomedical applications.

**Table 2:** Chemical structures bioavailability of the compounds and IC_50_ of them with Fibroblast Growth Receptor 1.

Compound	Chemical Structures	IC_50_ with FGFR1	Bioavailability per os
Fostamatinib	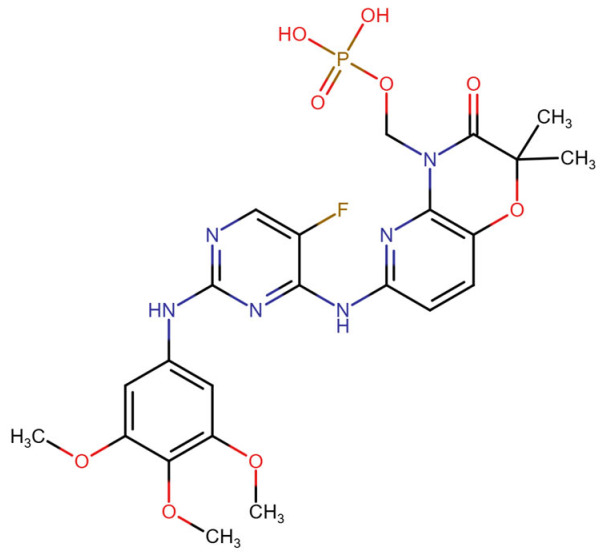	0.4 [87]	55% [88]
Erdafitinib	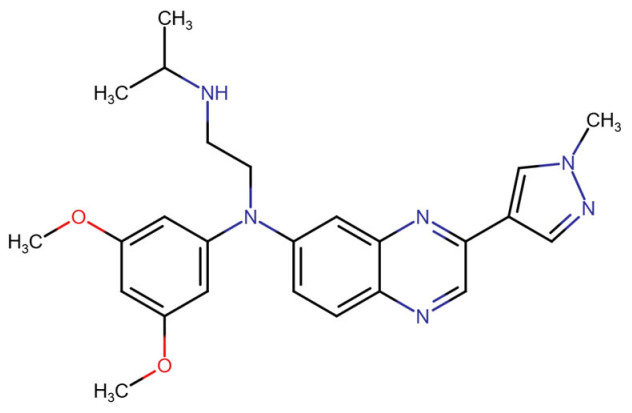	~2 nM [89]	60%
Futibatinib	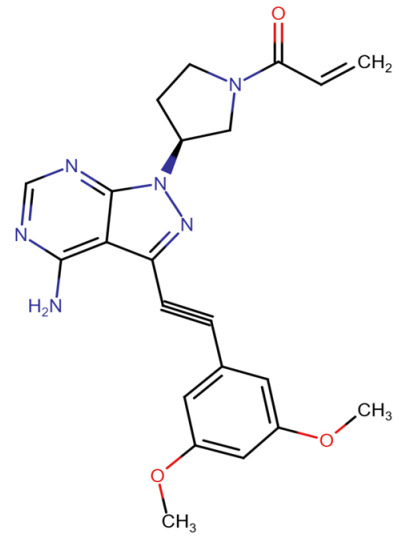	~1.4 nM [90]	79.8% [91]
Heparin	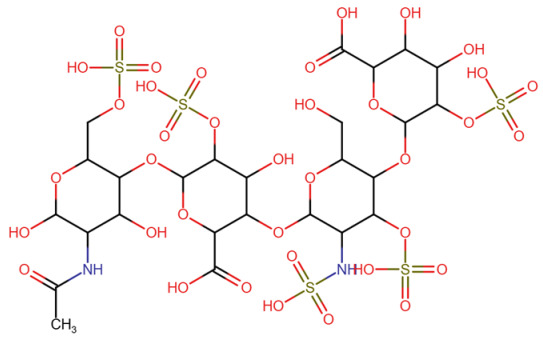	~63 nM [90]	Not reported
Infigratinib	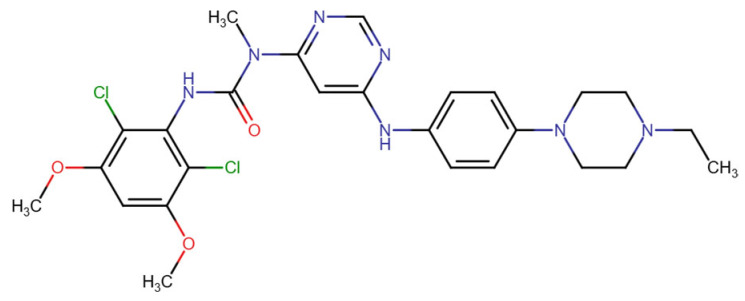	~1.1 nM [92]	~75% [93]
Lenvatinib	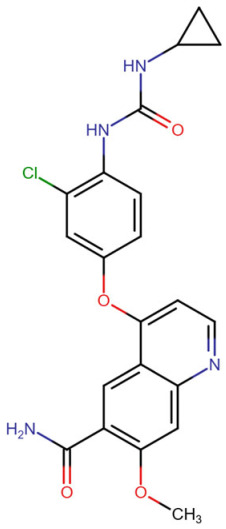	~46 nM [94]	85% [89]
Nintedanib	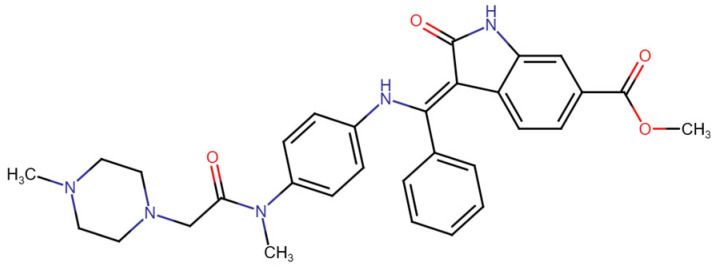	~69 nM	5%
Palifermin	C_721_H_1142_S_9_	Not reported	Not reported
Pemigatinib	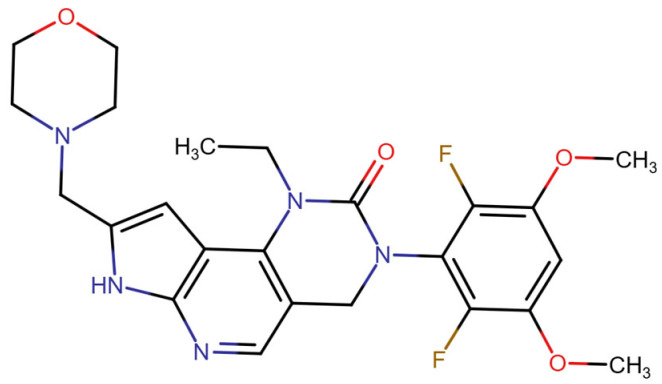	~0.4 nM	Not reported
Ponatinib	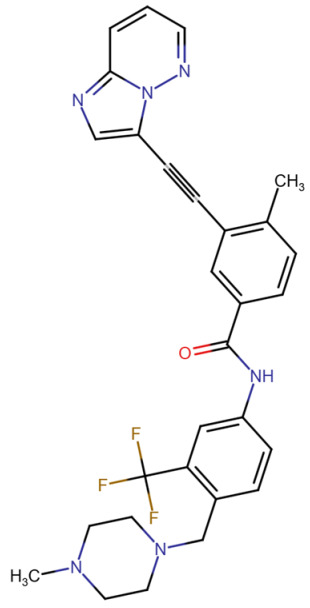	~2.2 nM [95]	54% [96]
Pralsetinib	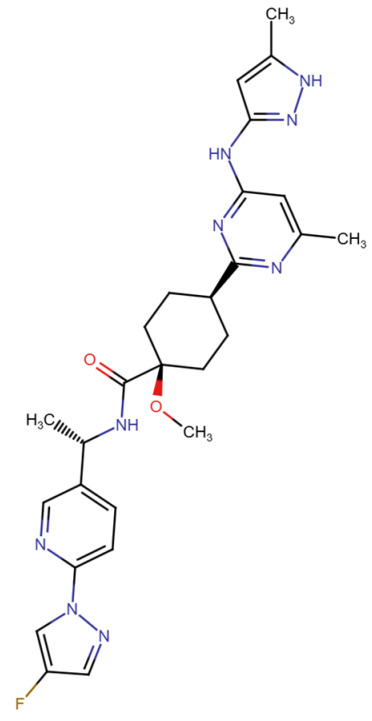	144–160 nM [97]	~70%
Regorafenib	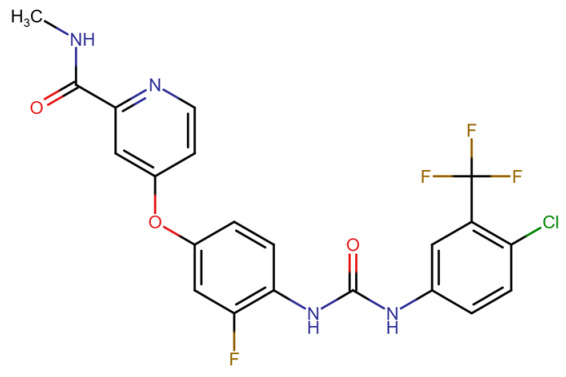	~202 nM [98]	69–83%
Romiplostim	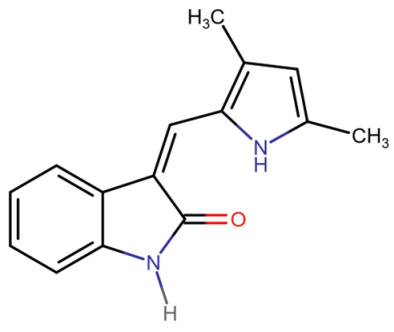	Not reported	~0%
Selpercatinib	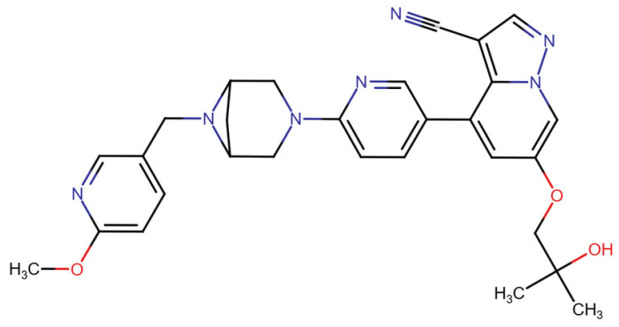	Not reported	73% [99]

#### 3.2.1. Fostamatinib

Activation of FGFR1 involves ligand-induced dimerization and transphosphorylation of tyrosine residues, especially within the activation loop and juxtamembrane regions. These phosphorylated tyrosines serve as docking sites for downstream signaling adaptors such as FRS2 and PLCγ [100]. Though fostamatinib is not a selective FGFR1 inhibitor, structural similarities among kinase domains suggest that off-target interactions may occur via these conserved motifs [101].

The binding of fostamatinib’s active metabolite R406 to kinase domains has been structurally characterized in complex with spleen tyrosine kinase (SYK) and other kinases. Although no crystal structure exists for FGFR1 bound directly to R406, structural comparisons using PDB ID: 4V05 (FGFR1 bound to AZD4547, a selective inhibitor) and PDB ID: 4PUZ (SYK bound to R406) reveal significant conservation of the ATP-binding cleft across these kinases [100,102]. R406 binds in the DFG-out conformation of SYK, forming two key hydrogen bonds with hinge region residues and hydrophobic contacts with residues lining the ATP pocket. By analogy, R406 is predicted to form similar interactions with FGFR1’s hinge region residues Met563 and Ala564, which correspond to the hydrogen bond acceptors seen in other kinase–R406 complexes. Additionally, critical FGFR1 residues involved in stabilizing Type II inhibitor binding include Lys514 (catalytic lysine), Glu531 (αC-helix), and Asp641–Phe642 (DFG motif), all of which contribute to ATP coordination and conformational dynamics [15].

Fostamatinib has been approved for immune thrombocytopenia due to its inhibitory effects on SYK-mediated Fc receptor signaling in macrophages [103]. However, broader kinase inhibition by R406 may impact pathways regulated by FGFR1, particularly in cancer, fibrosis, or tissue regeneration contexts [13]. Aberrant FGFR1 activity has been implicated in hematologic malignancies, and indirect suppression via multi-kinase inhibitors could contribute to therapeutic benefits [104]. Additionally, fostamatinib’s interference with PI3K/AKT and MAPK pathways, also downstream of FGFR1, indicates potential for pathway-level crosstalk and synergy in oncology applications [105].

#### 3.2.2. Erdafitinib

Erdafitinib is a potent, orally bioavailable, pan-FGFR tyrosine kinase inhibitor that targets fibroblast growth factor receptors 1 through 4 (FGFR1–4), with high affinity for FGFR1. The binding of erdafitinib to FGFR1 predominantly occurs at the intracellular tyrosine kinase domain, disrupting autophosphorylation and downstream signaling pathways such as PI3K-AKT and MAPK. Structural motifs essential for this interaction include the DFG (Asp-Phe-Gly) motif within the activation loop, which erdafitinib targets in its active (DFG-in) conformation. Erdafitinib inhibits FGFR1 by competing with ATP at the kinase domain, effectively suppressing ligand-mediated signaling involved in cell proliferation and survival [106,107].

Although a co-crystal structure of FGFR1 with erdafitinib has not been publicly resolved, comparative analysis with structurally similar FGFR inhibitors such as PDB ID: 4V05 allows inference of the binding mode. This inhibitor occupy the ATP-binding cleft of the FGFR1 tyrosine kinase domain, engaging key residues in the hinge region notably Ala564, Glu562, and Glu531 via hydrogen bonding. Erdafitinib, like 4V05, is presumed to bind the DFG-in conformation of FGFR1, interacting with residues in the P-loop (Gly485–Gly491), hinge region, and activation loop (Asp641–Phe642–Gly643). The central scaffold of erdafitinib is expected to form bidentate hydrogen bonds with the backbone of Ala564, a residue conserved across FGFR isoforms, anchoring the inhibitor in place. Hydrophobic contacts with residues such as Leu484, Val561, and Ala640 further stabilize the complex. These interactions facilitate effective kinase inhibition by preventing ATP binding and substrate phosphorylation, aligning with biochemical evidence from preclinical FGFR1 assays [108,109].

Erdafitinib has shown substantial clinical activity in tumors with FGFR1 alterations. In LUSC models, combining erdafitinib with STAT3 inhibitors enhances antitumor activity, supporting the importance of dual-pathway inhibition [110]. In the RAGNAR phase 2 study and the BLC2001 trial, erdafitinib elicited objective responses in patients with FGFR1–4-altered tumors, particularly urothelial carcinoma, demonstrating its efficacy in diverse histological settings [111,112]. Thus, the FGFR1–Erdafitinib interaction is not only structurally robust but also clinically significant, offering a promising strategy for treating FGFR-driven malignancies [106,107].

#### 3.2.3. Futibatinib

Futibatinib is a structurally novel, covalent inhibitor of FGFR1–4 that displays high affinity for key structural motifs within the ATP-binding pocket of these receptor tyrosine kinases. The drug forms an irreversible bond specifically with a conserved cysteine residue (Cys491 in FGFR2) located in the glycine-rich P-loop of the kinase domain, a region critical for ATP binding and catalysis. The glycine-rich loop’s conformational flexibility is thought to facilitate this binding mode, allowing futibatinib to trap multiple FGFR conformers [90,113].

Crystallographic and biochemical studies have elucidated the specific amino acid interactions involved in futibatinib binding to FGFRs. The covalent bond is formed with a conserved cysteine residue located within the P-loop of the kinase domain—specifically Cys488 in FGFR1 and Cys491 in FGFR2 [90,113]. This cysteine lies adjacent to the ATP-binding cleft and is part of a flexible glycine-rich loop that accommodates the acrylamide warhead of futibatinib. In addition to covalent bonding, futibatinib engages in key non-covalent interactions with residues in the hinge region, such as Ala564 and Glu565 in FGFR1, which stabilize the ligand within the active site. Hydrogen bonds with backbone atoms in the kinase hinge region, as well as van der Waals contacts with hydrophobic residues like Leu484 and Val561, further contribute to the high binding affinity and specificity [113,114].

Clinically, the irreversible inhibition of FGFR signaling by futibatinib translates into significant therapeutic activity and a favorable safety profile in patients with FGFR-driven tumors. Phase I–II trials demonstrated meaningful responses in intrahepatic cholangiocarcinoma (iCCA) patients harboring FGFR2 fusions, with an objective response rate of 42% and a median duration of response of 9.7 months. Hyperphosphatemia, an on-target effect related to renal FGFR inhibition, was the most common adverse event but was manageable with dietary and pharmacologic interventions. These findings underscore the biological and therapeutic relevance of futibatinib’s protein–ligand interaction features, supporting its clinical use in FGFR-aberrant malignancies [114,115,116].

#### 3.2.4. Heparin

FGFR1 contains three extracellular immunoglobulin-like (Ig) domains, with the heparin-binding domain localized between IgII and IgIII, a region rich in positively charged residues (lysines and arginines), which facilitates electrostatic interactions with heparin [117]. Structural modeling and mutational analyses have highlighted key residues such as Lys126, Arg121, and Asn28 in FGFR2 that directly interact with sulfated groups of heparin, enabling the formation of a stable complex with FGFR1 [118]. The ternary complex formed between FGFR1, FGF2, and heparin exhibits an electrostatic structure, with the negatively charged heparin nestled between the positively charged surfaces of FGFR2 and FGFR1 [119].

Several structural models and crystallographic studies have clarified the molecular details of the FGFR1–heparin interaction. PDB homology models and partial complexes have been constructed based on structures such as PDB ID: 1FQ9, which shows FGFR2 and FGFR1 bound to a heparin-derived hexasaccharide [118]. In these models, key positively charged residues on FGFR2—Lys27, Arg121, Lys126, Lys136, and Asn102—mediate electrostatic and hydrogen bond interactions with sulfated saccharide groups [118]. The FGFR1 ectodomain, particularly the IgII–IgIII linker region, contributes to heparin binding via conserved basic residues including Lys160, Arg163, and Lys168 [119]. The electrostatic model proposes that heparin is stabilized between two FGF2–FGFR1 complexes, enabling dimerization and receptor activation. Molecular modeling has further supported a 2:2:1 stoichiometry (2 FGFR1s: 2 FGF2s: 1 heparin), consistent with binding data and mutagenesis studies [118]. Despite the absence of a full PDB-resolved ternary structure, these data strongly support a mechanism in which heparin acts as a structural scaffold, orienting the proteins to facilitate receptor dimerization and signal initiation [120,121].

The FGFR1–heparin interaction is fundamental to effective FGF-mediated signal transduction and is implicated in numerous physiological and pathological processes, including cell proliferation, differentiation, angiogenesis, and cancer progression [117,121]. Blocking this interaction, such as with the IMB-R1 antibody that specifically targets the heparin-binding domain of FGFR1, has been shown to inhibit FGF2-induced cell growth and induce apoptosis in cancer cells [117]. The ability of heparin to facilitate dimerization and receptor activation supports its role as a crucial coreceptor in FGF signaling [119,122]. Therefore, understanding the structural basis of FGFR1–heparin interactions not only advances our knowledge of receptor biology but also opens avenues for targeted cancer therapies.

#### 3.2.5. Infigratinib

Infigratinib is a potent, ATP-competitive tyrosine kinase inhibitor with selectivity for FGFR1–3. It targets the highly conserved ATP-binding cleft within the intracellular kinase domain of FGFRs, crucial for receptor autophosphorylation and activation. Structurally, this interaction is mediated by infigratinib’s ability to bind key residues within the catalytic cleft of the tyrosine kinase domain, stabilizing the inactive conformation of FGFR and preventing downstream signaling [123]. Molecular docking and crystal structures deposited in the Protein Data Bank (e.g., PDB ID: 4V05) show that infigratinib forms hydrogen bonds with the hinge region (including Ala564 in FGFR1), hydrophobic interactions with residues such as Val561 and Leu630, and a critical interaction with Asp641 at the DFG motif of the activation loop [124]. These interactions underline the high binding affinity and selectivity of infigratinib for FGFR isoforms over other kinases.

The binding of infigratinib to FGFR1 has been extensively validated in various tumor models. Particularly in gliomas, activating point mutations in FGFR1 confer constitutive activation of the kinase and ligand-independent signaling. Infigratinib retains potent binding to these altered receptors, effectively suppressing their downstream oncogenic signaling [125]. The structural positioning of these mutations within the kinase domain preserves the binding cleft architecture, allowing infigratinib to maintain its affinity [126].

Clinically, the inhibition of FGFR signaling by infigratinib has profound implications in diseases. In multiple cancers, including glioblastoma and cholangiocarcinoma, FGFR fusions and mutations contribute to aberrant cell proliferation, survival, and angiogenesis [124]. Infigratinib suppresses these pathways, notably the MAPK, PI3K-AKT, and PLCγ signaling axes, by blocking receptor autophosphorylation. However, its efficacy may be modulated by tumor-specific co-mutations or pathway redundancies. For instance, concurrent mutations in PI3K pathway genes may necessitate combination therapies [126]. Moreover, in developmental biology, off-target effects of infigratinib at high doses highlight the relevance of FGFRs in normal morphogenesis and the need for careful dose titration in pediatric applications [123]. Thus, understanding the molecular underpinnings of FGFR–infigratinib interaction informs both precision oncology and safety profiling.

#### 3.2.6. Lenvatinib

Lenvatinib is a type V ATP-competitive inhibitor that binds to the kinase domain of FGFR1, stabilizing it in the DFG-out inactive conformation, thus preventing downstream signaling. Structural insights from PDB entry 5A46, which features FGFR1 bound to lenvatinib, reveal a conserved mode of interaction within the ATP-binding cleft. Critical interactions involve the hinge region residues Glu562 and Ala564, which form hydrogen bonds with the backbone of the inhibitor, anchoring it in place. The gatekeeper residue Val561, along with Leu484, Ala640, and Phe642, shapes the hydrophobic pocket that accommodates the aromatic core of lenvatinib. Additionally, Asp641 and Tyr653/Tyr654 within the activation loop contribute to conformational stability and are crucial for kinase activity modulation [127,128,129].

From a therapeutic perspective, lenvatinib’s inhibition of FGFR1 not only suppresses tumor growth but also improves the immune landscape in HCC. It reduces regulatory T cell (Treg) infiltration and PD-L1 expression by targeting FGFR1/FGFR4, enhancing the efficacy of immune checkpoint blockade with anti-PD-1 agents [130,131]. The combination of lenvatinib with agents like oxysophocarpine has shown synergistic effects by further downregulating FGFR1 expression and associated downstream signals [132]. This highlights the clinical relevance of FGFR1 inhibition in overcoming resistance mechanisms and improving patient outcomes, particularly in advanced HCC where FGFR1 overexpression correlates with poor prognosis and treatment failure [133].

#### 3.2.7. Nintedanib

Nintedanib is a small molecule that targets multiple receptor tyrosine kinases, including FGFR1, by competitively binding to the ATP-binding site of the receptor. FGFR1 is composed of an extracellular ligand-binding domain, a transmembrane domain, and an intracellular tyrosine kinase domain, which is the primary target for nintedanib. The ATP-binding pocket within this intracellular domain is crucial for receptor activation, and structural motifs such as the DFG (Asp-Phe-Gly) motif and hinge region residues are commonly involved in ligand binding. Although specific crystallographic structures of the FGFR1-nintedanib complex are limited, modeling and structural comparisons suggest nintedanib stabilizes the inactive conformation of FGFR1 by interacting with residues within the ATP-binding cleft, mimicking ATP hydrogen bonds and hydrophobic contacts [134,135].

Experimental data and in vitro studies indicate that key amino acid residues contributing to the binding of nintedanib include those located in the kinase domain of FGFR1 Lys514, Glu531, Val561, Glu562, and Asp641. These residues are critical for ATP coordination and contribute to the formation of hydrogen bonds and van der Waals interactions with nintedanib indolinone core and adjacent functional groups. Crystallographic modeling based on the FGFR1 tyrosine kinase domain structure PDB ID: 3C4F supports this interaction, showing that nintedanib occupies the ATP-binding cleft and stabilizes the DFG-in conformation. This type I binding mode effectively inhibits kinase activation and disrupts downstream signaling cascades such as MAPK/ERK [136,137,138].

Therapeutically, these interactions have significant implications for the treatment of diseases such as idiopathic pulmonary fibrosis and cancers like non-small cell lung cancer and esophagogastric adenocarcinoma. Nintedanib inhibition of FGFR1, along with VEGFR and PDGFR, results in suppression of angiogenesis, fibroblast proliferation, and tumor growth. In cancer, FGFR1 amplification is associated with aggressive behavior and resistance to therapy, making FGFR1 a critical target. The ability of nintedanib to maintain FGFR1 signaling suppression, even in resistant tumor subclones, offers a clinical benefit by delaying progression and overcoming resistance mechanisms, particularly when used in combination therapies [134,135,139].

#### 3.2.8. Palifermin

The interaction between fibroblast growth factor receptor 1 (FGFR1) and palifermin, a recombinant truncated form of keratinocyte growth factor (KGF/FGF7), is mediated through specific structural motifs on the extracellular domain of FGFR1. Binding primarily involves the immunoglobulin-like domains D2 and D3, as well as the D2–D3 linker region, which collectively determine ligand specificity and affinity [140]. These domains are both necessary and sufficient for ligand binding, whereas domain D1 and the D1–D2 linker act as autoinhibitory elements that modulate receptor activation. Structural studies have also highlighted the role of the alternatively spliced C′–E loop in domain III, which enhances the flexibility and specificity of FGF binding. This loop is particularly relevant in accommodating ligands like FGF1, which is known for its broad receptor promiscuity [140,141].

Crystallographic and biochemical analyses of FGFR–ligand complexes, including structures such as the FGFR3c–FGF1 complex (PDB: 1RY7), show that receptor activation requires not only ligand binding but also co-factors like heparin or heparan sulfate, which stabilize the formation of a ternary FGF–FGFR–heparin complex [142]. This interaction leads to receptor dimerization and subsequent transphosphorylation of intracellular tyrosine residues, initiating downstream signaling cascades. Key amino acid residues in the D2 and D3 domains of FGFR1—particularly Asn173, Glu176, and Lys160 in domain D2, and His254, Asn262, and Lys271 in domain D3—have been identified as essential contact points for FGF1 and related ligands [140]. Additionally, residues within the alternatively spliced C′–E loop, such as Pro252 and Ser254, contribute to the specificity of binding. Although palifermin binds with high specificity to FGFR2b via a similar interface, it has been observed to interact with FGFR1 under specific conditions, especially when FGFR1 is overexpressed or when cofactors modulate receptor conformation. Its reduced receptor promiscuity compared to FGF1 helps limit off-target activation, contributing to its safety profile in therapeutic applications [143].

Functionally, the FGFR1–palifermin interaction plays a significant role in epithelial tissue regeneration and cytoprotection. Palifermin activates several intracellular signaling pathways, including MAPK/ERK and PI3K/Akt, leading to enhanced epithelial cell survival, proliferation, and DNA repair [143]. Clinically, this translates into measurable therapeutic benefits. For instance, in patients with metastatic colorectal cancer undergoing fluorouracil-based chemotherapy, palifermin significantly reduced the incidence and severity of oral mucositis, thus improving tolerability and reducing chemotherapy dose modifications [144]. These effects validate the biological relevance of FGFR1–palifermin signaling and support its continued use and investigation in both oncologic supportive care and regenerative medicine [141,143].

#### 3.2.9. Pemigatinib

Pemigatinib, a potent and selective inhibitor, interacts primarily with FGFR1-3 by targeting the ATP-binding site, leading to inhibition of receptor autophosphorylation and downstream signaling. Pemigatinib’s molecular binding involves conserved residues in the kinase domain and exploits the DFG-in active conformation of FGFR1, which differentiates it from type II inhibitors like ponatinib that bind to a DFG-out inactive conformation [108,145].

**Figure 2:**
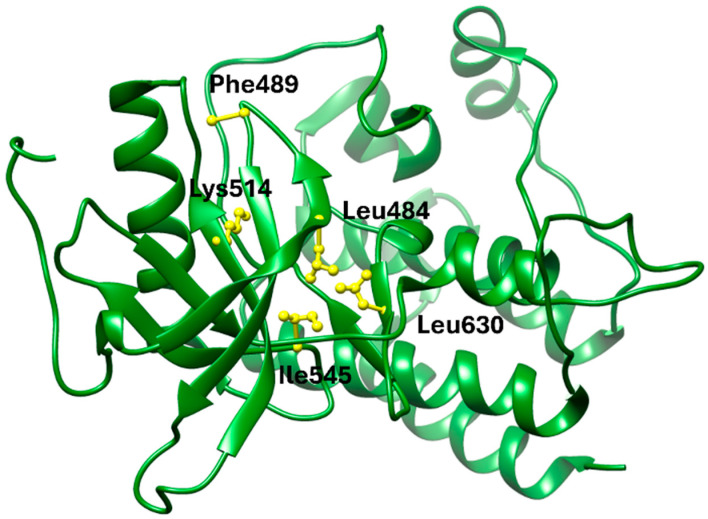
Crystal structure of the FGFR1 kinase domain (PDB ID: 4RWI) rendered in ribbon representation (green). For clarity, only chain A is shown. Amino acid residues of the FGFR1 receptor delineating the pemigatinib binding pocket (Leu484, Phe489, Lys514, Ile545, and Leu630) are highlighted in ball-and-stick form (yellow).

In terms of binding characteristics, crystal structural data and pharmacologic evaluations reveal that pemigatinib forms hydrogen bonds and hydrophobic contacts with specific residues such as Ala564, Glu531, and Phe642 within the kinase domain of FGFR1 [108]. These interactions stabilize the ligand within the ATP pocket and block the kinase activity by preventing ATP access. Structural insights into these interactions can be modeled using the crystal structure of FGFR1 bound to the selective inhibitor AZD4547, available in the Protein Data Bank under the accession code PDB: 4RWI, which shares a binding pocket of Leu484, Phe489, Lys514, Ile545, and Leu630 and highlights the critical residues in the ATP-binding cleft (Figure 2). Pemigatinib exhibits high selectivity for FGFR1–3, with IC_50_ values in 0.4–1 nM indicating that subtle differences in the amino acid environment surrounding the ATP-binding site contribute to binding specificity [146].

Biologically, the interaction between pemigatinib and FGFR1 has significant therapeutic implications. By inhibiting FGFR1 signaling, pemigatinib attenuates tumor cell proliferation, angiogenesis, and resistance mechanisms in FGFR-driven malignancies, including cholangiocarcinoma and urothelial cancers [147]. Pemigatinib’s selective engagement with FGFR delineates a pathway-specific oncologic intervention and offers a platform for precision oncology based on FGFR genotyping [148,149,150].

#### 3.2.10. Ponatinib

Ponatinib is a type II multikinase inhibitor that demonstrates strong binding affinity toward fibroblast growth factor receptor 1 (FGFR1), targeting its inactive DFG-out conformation. The binding pocket exploited by ponatinib includes the adenine region, a hydrophobic back pocket adjacent to the gatekeeper residue (Val561 in FGFR1), and a pocket created by the DFG-out conformation of the activation loop. Ponatinib’s rigid acetylene linker and trifluoromethylphenyl moiety facilitate van der Waals interactions and hydrogen bonding, securing the molecule within the ATP-binding cleft [108]. X-ray crystallography studies (PDB: 4V04) confirm that ponatinib binds FGFR1 in this DFG-out conformation, stabilizing an inactive state of the kinase and enabling high potency across FGFR isoforms [108,151].

At the amino acid level, key FGFR1 residues involved in ponatinib binding include the gatekeeper residue Val561, and other hydrophobic spine components such as Leu530, Met537, His544, and Phe565. These residues contribute to a tightly packed hydrophobic environment critical for type II inhibitor binding. Additionally, ponatinib forms hydrogen bonds with residues in the hinge region, notably Ala564, reinforcing its stable interaction [108]. This is clearly visualized in the crystal structure of the FGFR1–ponatinib complex (PDB ID: 4V04), where ponatinib occupies the ATP-binding cleft and extends into the hydrophobic pocket formed by the DFG-out conformation [151,152].

These interactions have critical implications for biological function and therapeutic efficacy. In models of FGFR1-driven malignancies such as 8p11 myeloproliferative syndrome (EMS) and FGFR1-fusion positive leukemias, ponatinib effectively suppresses downstream signaling via pathways such as PLCγ, STAT5, ERK1/2, and AKT, leading to reduced proliferation and apoptosis induction in vitro and in vivo [153,154]. Furthermore, in non-small cell lung cancer (NSCLC) cells overexpressing FGFR1, ponatinib demonstrated potent antiproliferative effects, suggesting therapeutic applicability in FGFR1-addicted solid tumors [155]. These preclinical outcomes underscore ponatinib’s capacity to act against both native and fusion-activated FGFR1 variants, providing a strong rationale for its continued investigation and clinical deployment in FGFR1-driven pathologies [156].

#### 3.2.11. Pralsetinib

The interaction between fibroblast growth factor receptor 1 (FGFR1) and the selective RET inhibitor pralsetinib exemplifies the cross-target promiscuity often observed in kinase-targeting therapies. Although pralsetinib was developed to inhibit RET-driven oncogenic signaling, its inhibitory activity extends to several other kinases, including FGFR1, TRK, VEGFR2, and PDGFRβ, due to structural similarities in the ATP-binding pockets [157,158]. FGFR1 contains a conserved tyrosine kinase domain that undergoes autophosphorylation upon ligand-induced dimerization, triggering downstream signaling cascades. These include the MAPK, PI3K-AKT, and PLCγ pathways, which are central to cell survival and proliferation [124].

Structurally, pralsetinib interacts with the FGFR1 kinase domain through key motifs near the ATP-binding cleft. These include the hinge region, gatekeeper residue, and DFG motif, which regulate kinase activity [108]. In FGFR1, critical amino acid residues involved in pralsetinib or related FGFR inhibitor binding include the gatekeeper residue Val561, hinge residue Glu562, and the conserved DFG motif residues Asp641–Phe643. These structural elements are essential for ATP binding and inhibitor selectivity. Relevant structural insights are drawn from PDB entry 4V05, which depicts FGFR1 in complex with the type II inhibitor ponatinib and illustrates a similar DFG-out conformation that could potentially be adopted by pralsetinib in off-target FGFR1 binding [108,124].

Clinically, the off-target inhibition of FGFR1 by pralsetinib may contribute to both therapeutic outcomes and adverse effects in RET-altered cancers. While RET fusions are the primary target in NSCLC and thyroid cancer, concurrent FGFR aberrations can exist, especially in aggressive tumors [159]. FGFR1 inhibition may provide additive or synergistic anticancer effects, particularly in tumors with FGFR1 amplification or dependency. However, this multi-target profile also introduces risks, such as hypertension and hepatotoxicity, potentially exacerbated by FGFR1’s role in vascular homeostasis [157].

#### 3.2.12. Regorafenib

Regorafenib, a type II multikinase inhibitor, preferentially binds FGFR1 in its DFG-out conformation, exploiting the hydrophobic back pocket adjacent to the ATP-binding site, a characteristic binding mode for type II inhibitors [128]. Structural analyses reveal that key motifs such as the gatekeeper residue (Val561 in FGFR1) and the molecular interaction network (involving residues Glu562, Asn546, and Lys638) contribute to the ligand selectivity and kinase inhibition, highlighting the importance of these domains in inhibitor design [13].

Although no crystallographic structure of an FGFR1–regorafenib complex has been resolved to date, mechanistic and structural insights suggest that regorafenib similarly targets the DFG-out conformation of the activation loop and interacts with key residues within the hydrophobic pocket adjacent to the ATP-binding site. These include the Phe residue of the DFG motif, Val561, and catalytic residues in the hinge region such as Glu562 and Lys638. Regorafenib’s broad-spectrum kinase inhibition includes potent activity against FGFR1 (IC_50_ ≈ 202 nM) [98,160]. Through stabilization of the inactive conformation of FGFR1, regorafenib is presumed to effectively inhibit autophosphorylation and downstream effector activation, contributing to its therapeutic efficacy.

Therapeutically, regorafenib’s interaction with FGFR1 is highly relevant in tumors where FGFR signaling drives oncogenesis or resistance to VEGF-targeted therapies. For instance, in colorectal and gastric cancers with FGFR2 amplification, regorafenib was shown to effectively inhibit receptor phosphorylation and downstream signaling, leading to reduced tumor proliferation and induced apoptosis in preclinical models [161]. Moreover, regorafenib has demonstrated survival benefits in hepatocellular carcinoma patients progressing on sorafenib, suggesting that FGFR inhibition plays a key role in its mechanism of action [162]. By targeting both angiogenic and oncogenic pathways, including FGFR1, regorafenib contributes to tumor microenvironment modulation and offers a therapeutic advantage in malignancies with activated FGFR1-2 signaling [160].

#### 3.2.13. Romiplostim

Romiplostim, although not a natural FGF ligand, acts on a separate receptor—the thrombopoietin receptor (MPL)—to stimulate megakaryocyte proliferation and platelet production [163]. However, FGFR1 remains central in mediating thrombopoiesis through the FGF1/FGFR1/PI3K/Akt/NF-κB axis, as demonstrated in models of radiation-induced thrombocytopenia where activation of FGFR1 upregulated this entire pathway, promoting platelet recovery [164].

At the structural level, crystallographic studies have elucidated the FGF2–FGFR1 interaction, with the dimeric complex structure resolved at 2.8 Å (PDB ID: 1CVS). The FGF2 ligand binds across both D2 and D3 domains of FGFR1, engaging residues within the β-sheets and the linker region between these domains [102,165]. FGFR1 residues Tyr653 and Tyr654 are key autophosphorylation sites, and their activation promotes downstream signaling. In contrast, romiplostim binds specifically to the MPL receptor using a peptibody scaffold composed of engineered thrombopoietin mimetic domains fused to an IgG Fc region [166]. Although romiplostim does not interact directly with FGFR1, cross-talk between FGFR1-driven and TPO/MPL-driven pathways has significant role, particularly in overlapping roles in megakaryocyte maturation and platelet production.

Romiplostim, by mimicking TPO and activating MPL, has shown high efficacy in treating chronic immune thrombocytopenia (ITP), with durable platelet responses and a favorable safety profile [163,166]. Meanwhile, activation of FGFR1, either endogenously by FGF1 or via pharmacological agents such as methylophiopogonanone A (MO-A), supports an alternative or complementary thrombopoietic strategy, especially in radiation-induced thrombocytopenia models [164,167].

#### 3.2.14. Selpercatinib

Selpercatinib is a highly selective small-molecule RET kinase inhibitor, yet it also exhibits limited off-target inhibition of FGFR1 at higher concentrations. FGFR1 is a receptor tyrosine kinase whose activation loop features a conserved Asp–Phe–Gly (DFG) motif, a crucial element in kinase regulation [168]. Selpercatinib is primarily designed for RET, its limited inhibition of FGFR1 suggests potential off-target activity through similar structural engagement of the activation loop, though at significantly reduced potency [169].

Crystallographic and biochemical analyses have shown that FGFR1 binds ponatinib at its ATP-binding cleft in the DFG-out conformation. In this context, key residues involved in inhibitor interaction include Val561, Ala564, and Tyr563, which stabilize the ponatinib–FGFR1 complex. Although selpercatinib is not a high-affinity binder of FGFR1, these conserved residues in the ATP-binding site might still facilitate weak interactions under high drug concentrations. The Protein Data Bank (PDB) entry associated with the FGFR1–ponatinib complex is 4V05, which captures the kinase in its inactive, DFG-out conformation stabilized by inhibitor binding [168]. The interaction with the activation loop, particularly Asp641 and the pH-sensitive ionization of this residue, contributes to the conformational equilibrium and the binding kinetics of type II inhibitors. This structural motif is critical for selective targeting and is likely involved in any off-target effects seen with selpercatinib when binding FGFR1 [170].

Dysregulation or overexpression of FGFR1 has been implicated in oncogenesis, particularly in cancers such as breast and lung carcinoma [171]. The limited but measurable inhibitory activity of selpercatinib on FGFR1 may contribute to off-target therapeutic or toxicological effects. However, its high selectivity for RET helps to minimize unintended inhibition of FGFR family members [172]. Clinically, selpercatinib’s success in treating RET-altered thyroid and non-small cell lung cancers is attributed to its potent and sustained inhibition of RET signaling [173,174]. Its therapeutic efficacy is reinforced by favorable pharmacokinetics and a manageable toxicity profile, distinguishing it from multikinase inhibitors that indiscriminately inhibit kinases such as FGFR1 and VEGFR2 [169].

#### 3.2.15. Sorafenib

Sorafenib, a multi-kinase inhibitor, interacts directly and indirectly with FGFR1 but exhibits limited specificity [175]. The binding mode of sorafenib, while not primarily optimized for FGFR1, can still impact this receptor via downstream off-target inhibition. Notably, sorafenib targets the phosphorylation of FGFR1, indirectly affecting downstream signaling cascades such as the MAPK/ERK, PI3K/AKT, and STAT3 pathways [176]. Structural analyses of FGFR1 reveal that key amino acid residues in the ATP-binding pocket (PDB IDs: 2FGI, 3GQL, 3C4F) such as Ala564, Glu562, and Cys488 are critical for ligand interaction and receptor activation [175]. Among them, Cys488, located within the glycine-rich loop of the kinase domain, is particularly notable as a target for covalent inhibitors due to its strategic position and low conservation across kinases. In the context of tumor angiogenesis, DJ-1 has been identified as a key secreted factor that promotes FGFR1 activation in endothelial cells by enhancing phosphorylation at these residues, thereby contributing to sorafenib resistance in hepatocellular carcinoma (HCC) [176]. Moreover, in malignant pleural mesothelioma tumor-initiating cells, the antiproliferative effect of sorafenib was associated with inhibition of FGFR1 phosphorylation at the kinase domain, leading to suppression of ERK and STAT3 activation following bFGF stimulation [177].

From a therapeutic perspective, FGFR1 overexpression and its downstream effector FRS2α correlate with reduced progression-free survival in patients treated with sorafenib, suggesting a role in intrinsic resistance mechanisms [178]. In hepatocellular carcinoma, FGFR1 activation promotes tumor-initiating cell enrichment and angiogenesis, contributing to acquired sorafenib resistance. Experimental models have shown that blocking FGFR signaling, either directly or via co-factors like DJ-1, can restore sorafenib sensitivity and suppress tumor growth [176,178]. These results emphasize the functional importance of FGFR1 in mediating therapeutic outcomes and advocate for the co-targeting of FGFR1 in cancers where resistance to sorafenib limits treatment efficacy.

### 3.3. GLP1R and GIPR

#### Tirzepatide

Tirzepatide is a synthetic 39-amino acid peptide designed to act as a dual agonist for both the glucose-dependent insulinotropic polypeptide receptor (GIPR) and the glucagon-like peptide-1 receptor (GLP1R). The GLP1R, a class B G protein-coupled receptor, contains two key domains essential for ligand binding: the extracellular N-terminal domain (NTD) and the transmembrane domain (TMD). The activation of GLP1R by peptide ligands, such as GLP-1 or tirzepatide, follows a two-domain model. In this model, the C-terminal region of the ligand initially interacts with the NTD, positioning the N-terminal domain of the ligand to engage deeply within the TMD, triggering receptor activation and downstream signaling [179,180]. Tirzepatide utilizes this structural framework to bind both incretin receptors, leveraging structural motifs from native GIP and exendin-4 to optimize binding efficacy and duration of action [181].

**Figure 3:**
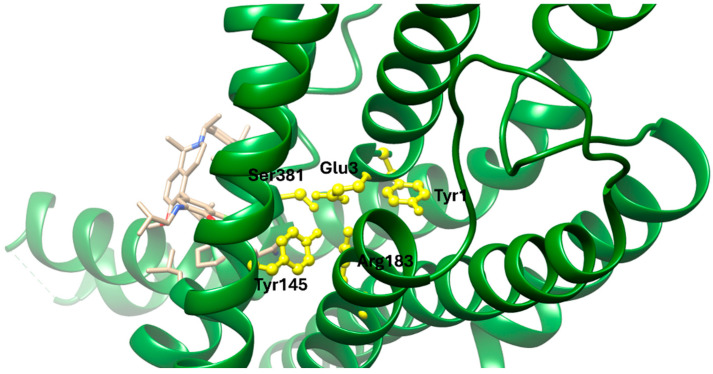
Cryo-EM structure of the human glucose-dependent insulinotropic polypeptide receptor (GIPR) bound to tirzepatide (PDB ID: 7RBT). For clarity, only chains F (GIPR) and G (tirzepatide) are shown, with all other chains omitted. The receptor is displayed in ribbon representation (green), and the peptide ligand is depicted in stick representation (beige). Key amino acid residues within the binding pocket interacting with tirzepatide (Tyr1, Glu3, Tyr145, Arg183, and Ser381) are highlighted in ball-and-stick representation (yellow), illustrating the critical recognition interface stabilizing the GIPR–tirzepatide complex.

The high-resolution cryo-EM structure of GLP1R bound to small-molecule agonists has illuminated key residues involved in ligand recognition and receptor activation. Based on Protein Data Bank entry PDB: 7RBT, and associated cryo-EM data, critical residues mediating binding include Glu138 and Leu141 in transmembrane domain 1 (TM1), Lys197 and Leu201 in TM2, Phe230 and Met233 in TM3, Thr298 in extracellular loop 2 (ECL2), and Arg380, Phe381, Leu384 and Phe385 in TM7 (Figure 3). Additionally, essential interactions in the N-terminal domain (NTD) are formed with Leu32, Trp33, Val36, and Gln37 [13]. Tirzepatide’s engineered hybrid sequence enables it to interact with both the NTD and TMDs, stabilizing the receptor in its active conformation and maximizing downstream cAMP-mediated signal transduction [172,179].

The dual receptor activity of tirzepatide results in enhanced insulinotropic effects, superior glycemic control, and greater weight reduction compared to GLP-1 receptor agonists alone. Clinical trials, particularly the SURPASS program, demonstrate tirzepatide’s dose-dependent superiority in lowering HbA1c and body weight [182,183]. Beyond metabolic improvements, tirzepatide has been associated with reductions in all-cause mortality and adverse cardiovascular and renal events compared to GLP-1R [180]. These therapeutic benefits are believed to stem from the synergistic activation of GLP1R and GIPR, with the latter contributing to enhanced β-cell function, insulin sensitivity, and central appetite regulation [181]. Thus, the structural and functional engagement of tirzepatide with GLP1R plays a pivotal role in its therapeutic success in type 2 diabetes and associated comorbidities.

**Figure 4:**
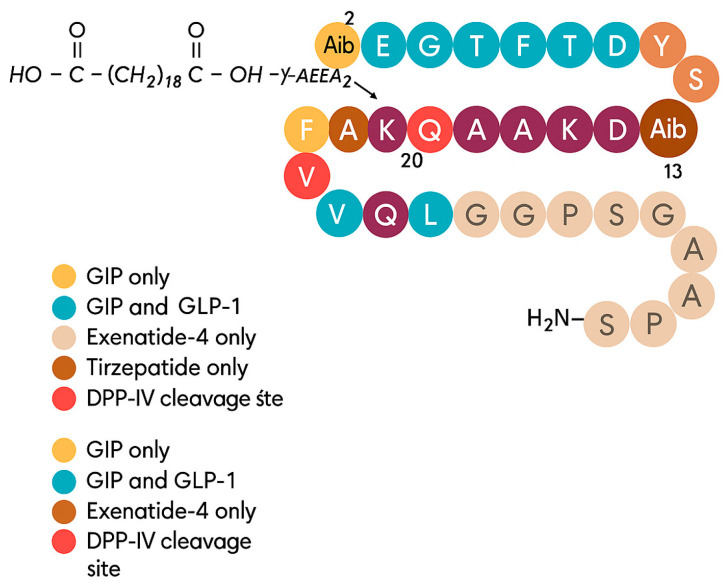
Tirzepatide (TZP) is a 39-amino acid peptide composed of both native and modified fragments derived from various incretins, including GIP, GLP-1, and Exendin-4 (excluding glucagon) (PDB ID: 7RGP). It features alpha-aminobutyric acid at positions 2 and 13, which contribute to its enhanced stability—resisting degradation by DPP-IV at position 2 and improving structural integrity at position 13. A 20-carbon fatty acid (eicosanedioic acid) is attached to a glutamic acid residue, and a double Ado unit is linked to the lysine at position 20. This modification enables TZP to bind to albumin, significantly prolonging its half-life to approximately five days [190].

Tirzepatide is a dual incretin receptor agonist that interacts with both the glucose-dependent insulinotropic polypeptide receptor (GIPR) and the glucagon-like peptide-1 receptor (GLP-1R), contributing to its therapeutic effects in type 2 diabetes (T2D) and obesity (Figure 4). Structurally, key binding domains include the N-terminal α-helix of tirzepatide that penetrates the transmembrane domain of both GIPR and GLP-1R. Cryo-EM studies show that tirzepatide mimics GIP in its interaction with GIPR, while displaying weaker interactions with GLP-1R due to diminished contact between its N-terminal Tyr1 and the GLP-1R core. This results in biased agonism at GLP-1R with reduced β-arrestin recruitment and receptor internalization [184,185]. These interactions enable tirzepatide to signal preferentially through cAMP generation while maintaining a long-acting profile via acylation with a C20 fatty diacid that binds albumin, enhancing its pharmacokinetics [186,187].

The structural determinants of tirzepatide’s binding were resolved through cryo-electron microscopy (cryo-EM) models—PDB: 7RBT for the GIPR–tirzepatide complex and GLP-1R–tirzepatide complex—revealing specific and differential residue-level interactions [184,187]. In the GIPR complex, tirzepatide residues Tyr1, Phe6, and Asp9 interact with GIPR residues Tyr1, Glu3, Tyr145, Arg183, and Ser381, forming stable hydrogen bonds and hydrophobic contacts within transmembrane helices 2, 3, and 5. These interactions create a compact and high-affinity ligand-receptor conformation, promoting efficient receptor activation. Conversely, at the GLP-1R, the same tirzepatide residues Tyr1, Phe6, and Asp9 show reduced interaction strength with GLP-1R residues Leu141, Glu364, and Lys197, leading to a more transient and weaker receptor engagement. This results in reduced receptor internalization and a signaling bias favoring cAMP generation over β-arrestin recruitment, ultimately leading to less desensitization. These structural nuances account for tirzepatide’s stronger and more stable activation of GIPR, while preserving a more tolerable signaling profile at GLP-1R, contributing to its superior metabolic efficacy and reduced gastrointestinal adverse effects compared to GLP-1 monotherapy.

These protein–ligand interactions have critical implications for biological function and therapeutic outcomes. The dual activation of GIPR and GLP-1R enhances insulin secretion, improves insulin sensitivity and reduces body weight. Tirzepatide’s biased signaling at GLP-1R avoids rapid receptor desensitization, allowing sustained insulinotropic action [185]. Clinically, this translates to superior glycemic control and weight loss compared to selective GLP-1R agonists [181,188]. Moreover, tirzepatide’s engagement with GIPR in adipose and pancreatic tissue contributes to enhanced metabolic flexibility and β-cell preservation, highlighting its advantage as a multi-target agent for obesity [186,189].

## 4. Protein-Ligand Interaction Features

### 4.1. PCSK9 (Proprotein Convertase Subtilisin/Kexin Type 9)

**Figure 5:**
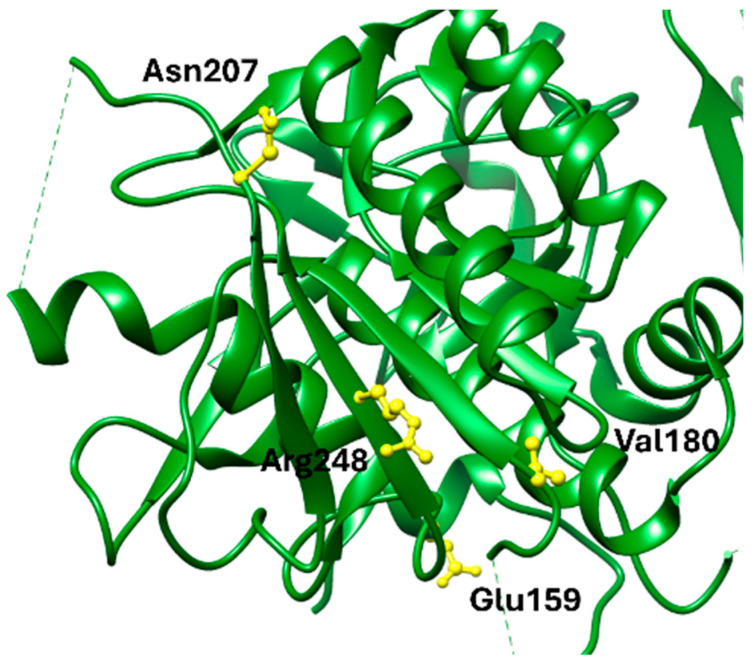
Crystal structure of human PCSK9 (PDB ID: 2P4E) shown in ribbon representation (green). All chains except the PCSK9 catalytic domain (chain E) are omitted for clarity, including the LDL receptor EGF-A domain (chain P). Key residues forming the binding pocket for alirocumab (Glu159, Val180, Asn207, Arg248) are depicted in ball-and-stick representation (yellow). This pocket corresponds to the functional interface targeted by alirocumab to block the PCSK9–LDLR interaction.

PCSK9 (PDB ID: 2P4E) is a protein that helps control cholesterol by breaking down Low-Density Lipoprotein (LDL) receptors, which normally remove “bad” LDL cholesterol from the blood. When PCSK9 is active, fewer receptors are available, and cholesterol levels go up. PCSK9 has three main parts: the prodomain (which helps with folding), the catalytic domain (which binds to LDL receptors), and the C-terminal domain (which helps with stability). The catalytic domain is the most important for binding and is the target of drugs like alirocumab (Figure 5) [17,191].

**Figure 6:**
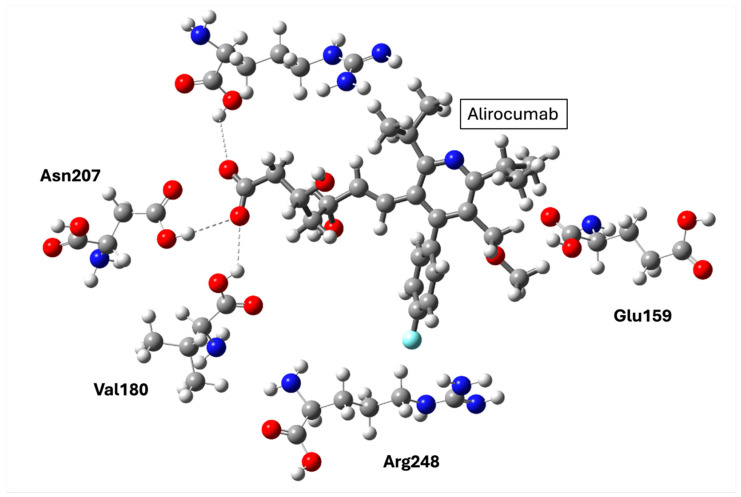
Docking pose of alirocumab in the catalytic domain of PCSK9 (PDB ID: 2P4E). The ligand (alirocumab) is depicted in stick representation, with interacting residues labeled by name and sequence number. Hydrogen bonds are observed with Asn207, Arg248, and Val180 (dotted lines), while ionic interactions involve Arg248.

In a study by Zuhier Awan et al. [192], using computational docking simulations, they assessed the binding affinity between alirocumab and PCSK9. The results indicated that alirocumab binds effectively to both forms of PCSK9, with a slightly stronger interaction observed with the mutant variant. Specifically, the total energy of binding (Etotal) was calculated to be −64.497 kJ/mol for the wild-type and −62.084 kJ/mol for the R496W mutant, suggesting a comparable binding strength in both cases. Specifically, alirocumab binds to the catalytic domain of PCSK9, the primary region involved in responsible for interacting with the LDL receptor (LDLR). Some key amino acids that form sone hydrogen bonds are Arg248 and Glu159 (Figure 6).

One other study by Sergio Fazio et al., they found that Evolocumab targets the catalytic domain of PCSK9, specifically binding to a region that overlaps with the epidermal growth factor-like repeat A (EGF-A) domain of the low-density lipoprotein receptor (LDLR) [193]. By doing so, it prevents PCSK9 from interacting with LDLR, thereby inhibiting the degradation of LDLR and promoting the clearance of LDL cholesterol from the bloodstream. Because evolocumab stops PCSK9, your body keeps more LDL receptors. This helps lower the LDL cholesterol, which can reduce the risk of heart disease [194].

Inclisiran is a small interfering RNA (siRNA) therapeutic that targets the messenger RNA (mRNA) of the PCSK9 gene in liver cells. By binding to a specific sequence on the PCSK9 mRNA, inclisiran facilitates its degradation through the RNA-induced silencing complex (RISC), thereby reducing the production of PCSK9 protein. This mechanism leads to increased availability of LDL receptors on hepatocyte surfaces, enhancing the clearance of LDL cholesterol from the bloodstream [195,196].

The specificity of inclisiran for PCSK9 mRNA has been demonstrated in preclinical studies, showing no significant suppression of genes with partial sequence homology to PCSK9.

This targeted approach allows for effective and sustained reduction in LDL cholesterol levels with infrequent dosing.

Evolocumab and alirocumab are medicines that bind to PCSK9 protein in your blood. This stops PCSK9 from breaking down the “good” LDL receptors, so the body can remove bad cholesterol faster. They work quickly but need injections every few weeks.

Inclisiran works inside the liver cells by blocking the instructions to make PCSK9. It takes a little longer to work but lasts much longer.

### 4.2. NF-κB (Nuclear Factor NF-Kappa-B p65 Subunit)

Dimethyl fumarate (DMF) affects the NF-κB pathway by targeting key proteins involved in inflammation. One major target is the p65 (RelA) subunit of NF-κB. DMF binds directly to a specific cysteine residue (Cys38) on p65, which blocks the protein from moving into the cell nucleus and prevents it from attaching to DNA and activating inflammation-related genes [197]. Another important protein affected by DMF is thioredoxin-1 (Trx1), a redox regulator that helps maintain the activity of NF-κB by keeping certain cysteine residues in a reduced, active state. DMF inhibits Trx1, which disrupts this process and reduces NF-κB activity, leading cells toward apoptosis or necroptosis [198]. In addition, DMF influences the movement of NF-κB into the nucleus. Normally, NF-κB is held in the cytoplasm by a protein called IκBα, which is degraded when the cell is stimulated, allowing NF-κB to enter the nucleus. DMF does not prevent the degradation of IκBα, but it still manages to block the nuclear entry of NF-κB, effectively silencing the expression of pro-inflammatory genes [199].

Moreover, in a study by Jin-A. Shin et al., they studied a special type of glucosamine-based compound called NK-4 to see how it affects inflammation [200]. The key part is how NK-4 affects NF-κB, a protein complex that controls the genes involved in inflammation. It prevents NF-κB from sticking to DNA, which is necessary to activate inflammatory genes. So in short, NK-4 reduces inflammation by blocking NF-κB from turning on inflammatory genes, even though it lets NF-κB move freely inside the cell.

**Figure 7:**
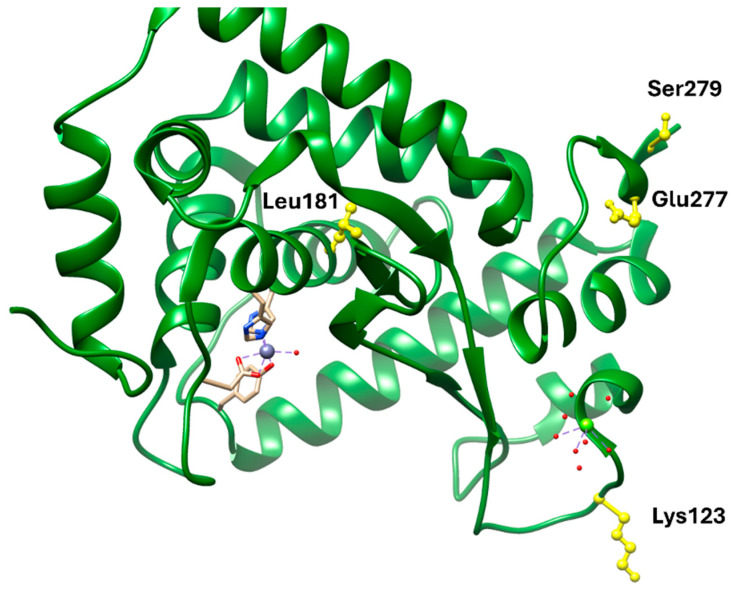
Crystal structure of the NF-κB p65 subunit in complex with parthenolide (PDB ID: 4Q3J) shown in ribbon representation (green). The covalently bound parthenolide molecule is displayed in stick representation (beige). Key amino acid residues forming the binding pocket and interacting with parthenolide (Lys123, Leu181, Glu277, Ser279) are highlighted in ball-and-stick representation (yellow). These residues constitute the functional interaction site through which parthenolide exerts its inhibitory effect on NF-κB activity.

The crystal structure of the NF-κB p65 subunit complexed with parthenolide (PDB ID: 4Q3J) reveals the molecular basis of its inhibitory interaction. As shown in Figure 7, the protein is depicted in ribbon representation (green), while parthenolide is displayed in stick form (beige) covalently bound within the binding pocket. Critical amino acid residues, including Lys123, Leu181, Glu277, and Ser279, are highlighted in ball-and-stick representation (yellow). These residues form the functional interaction interface responsible for stabilizing parthenolide binding and mediating its inhibitory effect on NF-κB activity, ultimately interfering with the transcriptional regulation controlled by this signaling pathway.

**Figure 8:**
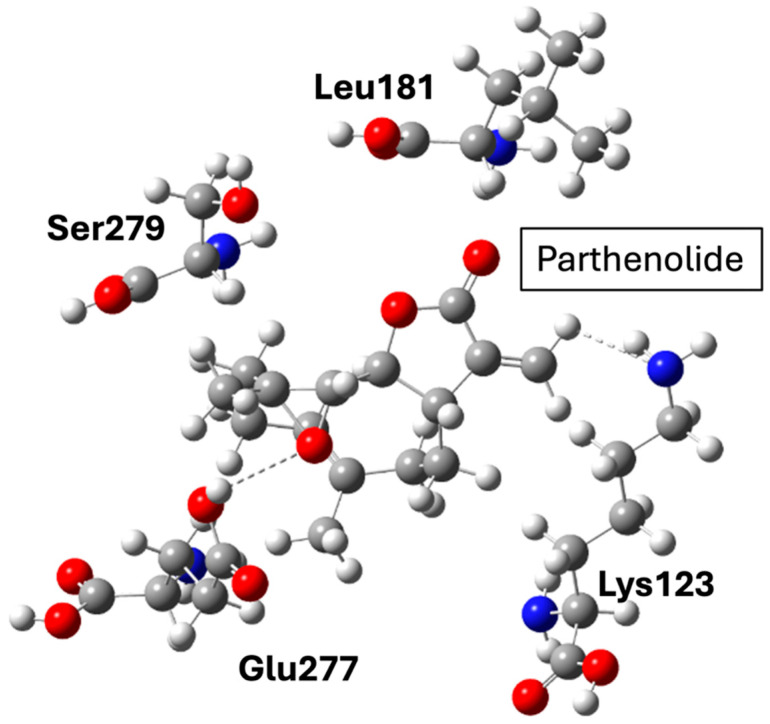
Docking pose of parthenolide in the active site of NF-κB (PDB ID: 4Q3J). The ligand (parthenolide) is depicted in stick representation, with interacting residues labeled by name and sequence number. Hydrogen bonds (dotted lines) are formed with Glu277 and Lys123.

Furthermore, in a study by Mona Dawood et al., scientists found that parthenolide blocks inflammation by stopping a key step in the NF-κB pathway [201]. Parthenolide binds to a protein called IKKβ, which is normally responsible for activating NF-κB. It attaches to a part of IKKβ (Figure 8). As a result, NF-κB becomes inactive and cannot go into the cell nucleus to activate inflammation or cancer-related genes. In short, parthenolide turns off NF-κB by sticking to a key spot on IKKβ, keeping inflammation and cancer cell survival genes from being activated.

Continuing to this enzyme, it was found that sulfasalazine works by blocking a protein called IKKβ (part of the IKK complex). This protein normally adds a chemical tag (phosphate) to another protein called IκBα, which causes IκBα to break down. Once IκBα breaks down, NF-κB is free to move into the nucleus and turn on inflammation genes. Specifically, sulfasalazine sticks to the energy site on IKKβ and shuts it down, keeping NF-κB inactive [202].

One other example of inhibitor to NFkB is tyloxapol. Although the exact binding site is not fully characterized, but it clearly works upstream in the NF-κB pathway. This occurs by blocking the signals that lead to IκBα breakdown, which helps lower inflammation [203].

In a study of Panagiotis Ntavakouras et al. [204], it was shown that Q3 which is a quinoline derivative, inhibits stronger NF-kB when it is complex with DNA. Molecular dynamics simulations further supported these findings. The root mean square deviation (RMSD) analysis showed that the NF-κB/Q3 complex maintained stability throughout the simulation, indicating strong and sustained binding. These computational analyses suggest that Q3 is a promising candidate for further development as an NF-κB inhibitor [204].

### 4.3. NLRP3 (NACHT, LRR and PYD Domains-Containing Protein 3)

In a study from Emine Erdag et al. [205], molecular docking simulations were performed to evaluate the binding affinities of several ligands targeting the NLRP3 inflammasome, which are key mediators in endodontic inflammation. The docking scores for the tested ligands ranged from −5.1 to −11.8 kcal/mol, indicating a spectrum of binding strengths. Among the compounds evaluated, CY-09 emerged as a potent inhibitor of the NLRP3 inflammasome, showing strong binding affinity and favorable inhibition constants. Critical amino acids that CY-09 interacted with NLRP3 are shown in Table 3.

**Figure 9:**
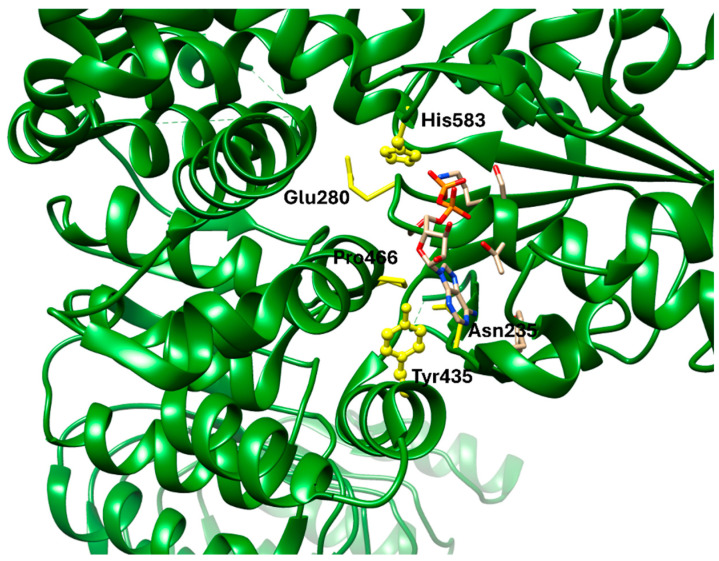
Crystal structure of the NACHT domain of human NLRP3 (PDB ID: 5IRM) shown in ribbon representation (green). For clarity, only chain A is displayed, while chain C is omitted since the structure is homodimeric. Key amino acid residues forming the ligand-binding pocket (Glu280, Asn255, Tyr435, Pro466, and His583) are highlighted in ball-and-stick representation (yellow). The bound ADP molecule is shown in stick representation, positioned within the binding site, illustrating the critical molecular interactions stabilizing the NLRP3–ADP complex.

The crystal structure of the NACHT domain of human NLRP3 (PDB ID: 5IRM) provides insight into the molecular interactions underlying its nucleotide-binding activity. As illustrated in Figure 9, the domain is presented in ribbon representation (green), with key residues that form the ligand-binding pocket—Glu280, Asn255, Tyr435, Pro466, and His583—highlighted in ball-and-stick form (yellow). The ADP molecule, depicted in stick representation, is bound within this pocket, demonstrating how these residues contribute to stabilizing the NLRP3–ADP complex. This interaction is essential for the conformational regulation of NLRP3 and plays a critical role in its activation and function within the inflammasome signaling pathway.

Finally, in a study of Nedra Mekni et al. [206], molecular docking and dynamics simulations were used to investigate how the inhibitor MCC950 binds to the NLRP3 domain. Docking showed that MCC950 binds more strongly in the presence of adenosine triphosphate (ATP), with a docking score of −8.850 kcal/mol, compared to −6.044 kcal/mol without ATP. Key interactions involved hydrogen bonds with residues like Ser271, Pro281, and Asn284. Molecular dynamics simulations confirmed the stability of the MCC950–NLRP3 complex over time, supporting the potential of these binding sites for future inhibitor design.

### 4.4. Broader Translational Axis: FXR and ACL Targets

Beyond the six primary molecular targets analyzed in this review, additional therapeutics such as obeticholic acid and bempedoic acid extend the translational scope of cardiometabolic intervention. Obeticholic acid, a selective farnesoid X receptor (FXR) agonist, improves bile acid metabolism and exerts downstream effects on glucose and lipid homeostasis, while also attenuating hepatic inflammation and fibrosis. Clinical evaluation in primary biliary cholangitis demonstrated significant biochemical and histological improvements, underscoring the therapeutic potential of FXR activation in metabolic and inflammatory disorders [207]. Similarly, bempedoic acid, an ATP-citrate lyase (ACL) inhibitor acting upstream of HMG-CoA reductase, provides LDL-cholesterol reduction and cardiovascular benefit in patients intolerant to statins. The CLEAR Outcomes trial confirmed its efficacy and safety, establishing bempedoic acid as a non-statin alternative for long-term cardioprotection [208]. Together, these agents exemplify how modulation of auxiliary metabolic pathways complements incretin- and lipid-focused therapeutics to achieve integrated cardiometabolic benefits.

### 4.5. Mechanistic Network and Crosstalk Among Cardiometabolic Targets

Cardiometabolic signaling involves an intricate and highly coordinated network that integrates incretin, lipid, and inflammatory pathways into a unified regulatory framework. Activation of GLP-1R and GIPR stimulates AMPK and PI3K–AKT cascades, leading to enhanced glucose uptake, improved mitochondrial efficiency, and increased insulin sensitivity. These metabolic effects converge with FGFR1/β-Klotho–mediated MAPK signaling, which further promotes fatty acid oxidation, adipocyte differentiation, and overall energy expenditure. Through these complementary mechanisms, incretin and FGF pathways maintain the dynamic balance between nutrient sensing and metabolic homeostasis.

Conversely, PCSK9 operates at the intersection of lipid metabolism and inflammation by regulating LDL receptor (LDLR) turnover and cholesterol transport. Elevated PCSK9 levels not only impair hepatic LDL clearance but also amplify vascular inflammation through NF-κB and NLRP3 activation in macrophages and endothelial cells. Inhibition of PCSK9 therefore exerts secondary anti-inflammatory effects and contributes to atheroprotection beyond lipid lowering.

NF-κB and NLRP3 represent central nodes of the innate immune network that drive cytokine production (IL-1β, IL-6, TNF-α) and insulin resistance under metabolic stress. Their overactivation establishes a pro-inflammatory milieu that perpetuates cardiometabolic dysfunction. Importantly, upstream modulation of GLP-1R, GIPR, or FGFR1 signaling can suppress these inflammatory nodes via AMPK-dependent inhibition of IKK activity and attenuation of inflammasome assembly. This reciprocal regulation illustrates how metabolic cues can actively repress inflammation, while chronic inflammatory signaling, in turn, perturbs metabolic control.

Taken together, these interlinked pathways define a systems-level landscape in which glucose and lipid metabolism, energy expenditure, and inflammatory tone are tightly interconnected. Understanding this mechanistic crosstalk provides the conceptual basis for the rational design of multi-target ligands capable of restoring metabolic equilibrium and cardioprotection. This section conceptually summarizes the mechanistic interactions among incretin, lipid, and inflammatory targets, providing an integrated view equivalent to a systems-level diagram.

## 5. Future Perspectives

The simultaneous modulation of NFκB, PCSK9, NLRP3, FGFR1, GLP1R, and GIPR is an appealing yet demanding route toward next-generation therapeutics. Inspection of the ligand-recognition pockets across the relevant structures—NFκB p65 with parthenolide (4Q3J: Lys123, Leu181, Glu277, Ser279), PCSK9 at the alirocumab epitope (2P4E: Glu159, Val180, Asn207, Arg248), NLRP3 NACHT at the ADP site (5IRM: Glu280, Asn255, Tyr435, Pro466, His583), FGFR1 kinase with pemigatinib (4RWI: Leu484, Phe489, Lys514, Ile545, Leu630), GLP1R at the exenatide pocket (6X18: Lys197, Tyr205, Gln210, Gln211, Trp214, Gln234, Thr298, Arg299, Asn300), and GIPR at the tirzepatide pocket (7RBT: Tyr1, Glu3, Tyr145, Arg183, Ser381)—indicates that a successful hybrid must balance complementary interaction motifs.

First, aromatic moieties (e.g., phenyl, indole, heteroaryl) should be incorporated to engage π–π and edge-to-face contacts with Phe489 (FGFR1), Tyr435 (NLRP3), Tyr205/Trp214 (GLP1R), and Tyr145 (GIPR). Second, hydrophobic substituents (alkyl/cycloalkyl) can occupy nonpolar regions defined by Val180 (PCSK9) and Leu484, Ile545, Leu630 (FGFR1). In parallel, polar functions (amide, urea, hydroxyl, heteroaromatic nitrogens) should be positioned to form directional hydrogen bonds with Gln210/Gln211/Gln234 and Asn300 (GLP1R), Ser279 (NFκB), Ser381 (GIPR), Glu159 (PCSK9), and His583/Asn255 (NLRP3). Because ligand recognition also involves cationic residues—Lys123 (NFκB), Lys197 (GLP1R), Lys514 (FGFR1), Arg248 (PCSK9), Arg299 (GLP1R), Arg183 (GIPR)—and anionic residues—Glu277 (NFκB), Glu280 (NLRP3), Glu159 (PCSK9), Glu3 (GIPR)—the hybrid scaffold should present both a basic center (secondary/tertiary amine) and an acidic center (carboxylate or tetrazole) on separate arms to enable context-dependent salt-bridge formation without intramolecular charge repulsion (Table 4).

**Figure 10:**
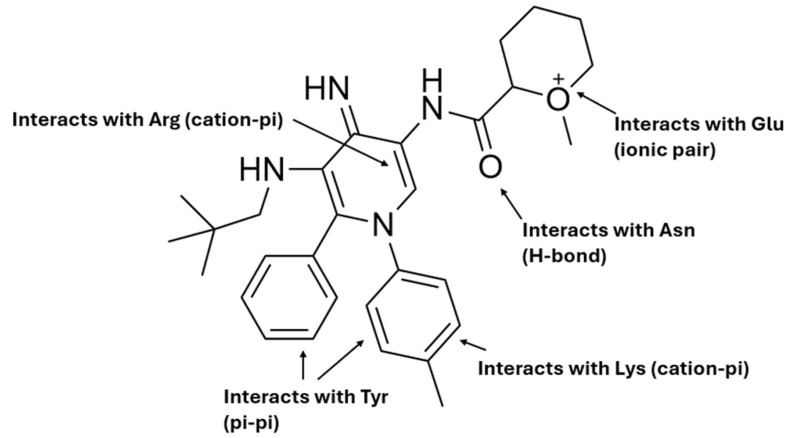
Schematic representation of the proposed hybrid molecule and its predicted interactions with key amino acid residues commonly found in the binding pockets of the six studied receptors. The phenyl substituent participates in π–π stacking with Tyr, while the central aromatic scaffold establishes cation–π interactions with Arg and Lys. The cationic side chain forms an ionic pair with Glu, and the amide group acts as a hydrogen bond donor/acceptor interacting with Asn. This structural design highlights the rational mapping of distinct molecular moieties to conserved amino acid residues across the receptor binding sites.

From a design perspective, a modular architecture is advised: a rigid or semi-rigid heteroaromatic core as the anchor, bearing two or more substituent arms that project into distinct subpockets, connected (where needed) by a flexible linker of ~3–6 bonds to accommodate cross-target variability while maintaining compactness. To preserve drug-like behavior, aim for MW 380–600 Da, topological polar surface area < 140 Å^2^, and 3–8 rotatable bonds; judicious bioisosteres (e.g., tetrazole ↔ carboxylate) can further refine stability, permeability, and target engagement across these pockets (Figure 10).

## 6. Conclusions

Cardiometabolic diseases constitute a major global health burden, demanding strategies that move beyond single-target interventions. In this review we examined six pivotal molecular targets (GLP-1R, GIPR, FGFR1, PCSK9, NF-κB, and NLRP3) which collectively shape glucose regulation, lipid metabolism, inflammation, and cardiovascular remodeling. The progress achieved in structural biology and ligand discovery underscores that therapeutic efficacy is fundamentally driven by the quality of protein–ligand interactions. A deeper mechanistic and structural understanding of these interactions is essential for accelerating the rational design of safer and more effective multi-target agents.

Emerging therapies such as dual and multi-receptor agonists, monoclonal antibodies, and small-molecule inhibitors demonstrate clear advantages over traditional monotherapies by combining metabolic control with cardioprotective and anti-inflammatory effects. Yet, challenges remain: incomplete structural data, the potential for off-target or pleiotropic effects, and limited oral bioavailability in many candidates. Addressing these limitations will require integrating structural and mechanistic insights with systems biology and translational research. Such an approach will be critical for translating molecular knowledge of these six targets into clinically robust therapies that can mitigate the complex burden of cardiometabolic disease.

Despite significant advances in structural biology and molecular modeling, current structure-based approaches in cardiometabolic drug discovery still face important limitations. The intrinsic conformational flexibility of G protein-coupled receptors (GPCRs), the dynamic nature of protein–ligand interfaces, and the complexity of multi-domain targets such as FGFR1 or inflammasome components restrict the predictive power of static crystallographic and docking models. Furthermore, off-target interactions and incomplete characterization of membrane microenvironments continue to pose challenges for safety and translational reliability. Future progress will depend on the integration of next-generation computational and experimental tools capable of capturing the full dynamic landscape of these systems. Artificial intelligence and machine learning are increasingly applied to accelerate ligand screening, predict binding conformations, and design multitarget molecules with optimized pharmacokinetic properties. Parallel advances in cryo-electron microscopy (cryo-EM) and hybrid modeling now allow high-resolution visualization of membrane protein complexes under near-physiological conditions, complementing traditional crystallography. Together, these emerging strategies are expected to transform structure-based drug design into a more predictive, systems-level discipline capable of delivering safer and more effective cardiometabolic therapeutics.

The reviewed ligands display varying degrees of specificity depending on their molecular scaffolds and binding site features. Peptidic agonists targeting GLP-1R and GIPR demonstrate high selectivity with minimal cross-reactivity among class B GPCRs, whereas certain kinase inhibitors such as those acting on FGFR1 may exhibit partial overlap across isoforms. Competitive binding among different ligands for the same receptor is theoretically possible but rarely clinically significant due to receptor biasing and optimized dosing. Moreover, protein-mediated disruption or degradation of ligand–target complexes is limited, as most therapeutic ligands possess strong binding affinities and structural modifications that enhance metabolic stability.

## Figures and Tables

**Table 3:** Critical amino acid residues of NLRP3-PYD peptide active site interacting with NLRP3.

Inhibitor	Amino Acids
CY-09	ASN235, PRO466, TYR435, HIS583, GLU280

**Table 4:** Common amino acids in the binding pockets of the six receptors (GLP-1R, FGFR1, GIPR, NF-ΚB, PCSK9, NLRP3), grouped by residue type.

Residue Type	Common Amino Acids	Receptors Involved
Aromatic	Tyr	GLP-1R, GIPR, NLRP3
Basic	Arg, Lys	GLP-1R, PCSK9, GIPR, NF-κB, FGFR1
Acidic	Glu	PCSK9, NF-κB, NLRP3, GIPR
Polar	Asn	GLP-1R, PCSK9, NLRP3

## Data Availability

This article does not contain any original data. The content is based entirely on previously published literature, which is cited throughout the manuscript.
