# Correction: Petsas et al. Protein–Ligand Interactions in Cardiometabolic Drug Targets: Focus on Weight Loss and Cardioprotection. *Molecules* 2025, *30*, 4240

**DOI:** 10.3390/molecules31040609

**Published:** 2026-02-10

**Authors:** Errikos Petsas, Despoina P. Kiouri, Nikitas Georgiou, Gerasimos Siasos, Thomas Mavromoustakos, Christos T. Chasapis

**Affiliations:** 1 Laboratory of Organic Chemistry, Department of Chemistry, National and Kapodistrian University of Athens, 11571 Athens, Greece; errpets@chem.uoa.gr (E.P.); despoina.kiouri.99@gmail.com (D.P.K.); nikitasgalleti93@hotmail.com (N.G.); tmavrom@chem.uoa.gr (T.M.); 2 Center for Interdisciplinary Biosciences, Technology and Innovation Park, P.J. Safarik University in Kosice, 040 01 Kosice, Slovakia; 3 3rd Department of Cardiology, Thoracic Diseases General Hospital Sotiria, Medical School, National and Kapodistrian University of Athens, 11527 Athens, Greece; gsiasos@med.uoa.gr

## Error in Figure 10

In the original publication [1], an incorrect chemical formula was shown in Figure 10. The corrected Figure 10 is provided below. The authors state that the scientific conclusions are unaffected. This correction was approved by the Academic Editor. The original publication has also been updated.

**Published version of Figure 10:**

**Figure 10:**
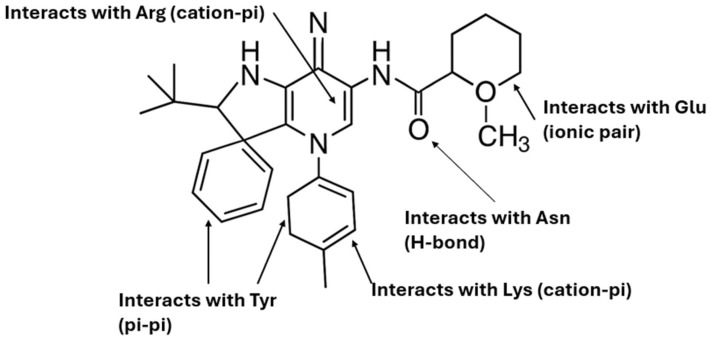
Schematic representation of the proposed hybrid molecule and its predicted interactions with key amino acid residues commonly found in the binding pockets of the six studied receptors. The phenyl substituent participates in π–π stacking with Tyr, while the central aromatic scaffold establishes cation–π interactions with Arg and Lys. The cationic side chain forms an ionic pair with Glu, and the amide group acts as a hydrogen bond donor/acceptor interacting with Asn. This structural design highlights the rational mapping of distinct molecular moieties to conserved amino acid residues across the receptor binding sites.

**Corrected version of Figure 10:**

**Figure 10:**
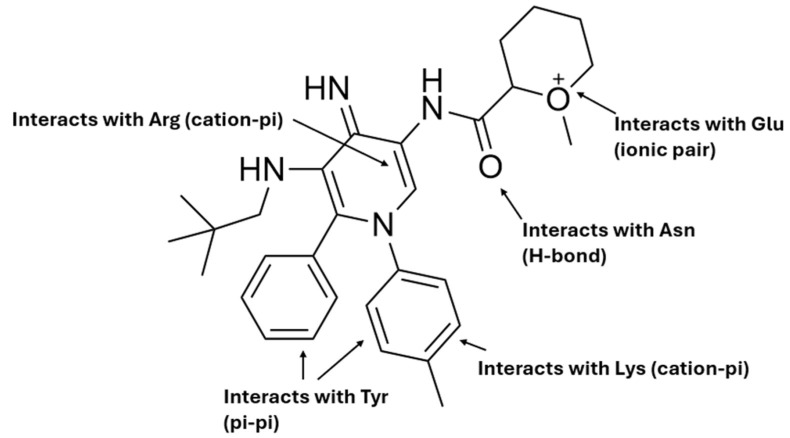
Schematic representation of the proposed hybrid molecule and its predicted interactions with key amino acid residues commonly found in the binding pockets of the six studied receptors. The phenyl substituent participates in π–π stacking with Tyr, while the central aromatic scaffold establishes cation–π interactions with Arg and Lys. The cationic side chain forms an ionic pair with Glu, and the amide group acts as a hydrogen bond donor/acceptor interacting with Asn. This structural design highlights the rational mapping of distinct molecular moieties to conserved amino acid residues across the receptor binding sites.

## Error in Table 2

In the original publication [1], graphical inaccuracies in the chemical formulas were present in Table 2. The corrected Table 2 is provided below. The authors state that the scientific conclusions are unaffected. This correction was approved by the Academic Editor. The original publication has also been updated.

**Published version of Table 2:**

**Table 2:** Chemical structures bioavailability of the compounds and IC_50_ of them with Fibroblast Growth Receptor 1.

Compound	Chemical Structures	IC_50_ with FGFR1	Bioavailability per os
Fostamatinib	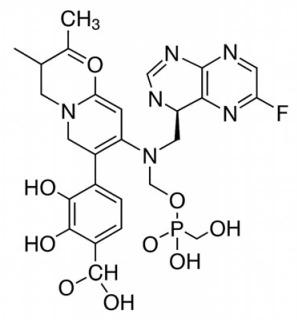	0.4 [87]	55% [88]
Erdafitinib	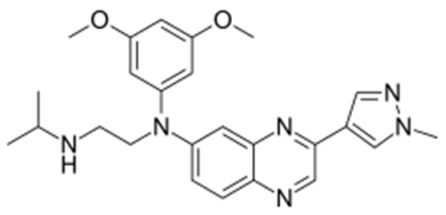	~2 nM [89]	60%
Futibatinib	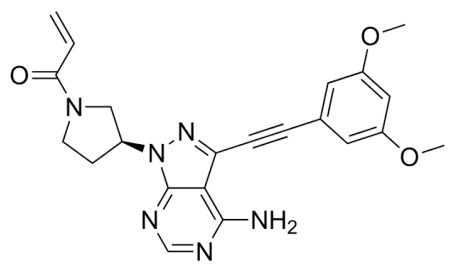	~1.4 nM [90]	79.8% [91]
Heparin	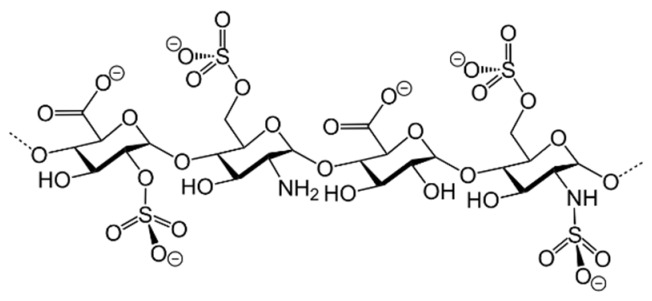	~63 nM [90]	Not reported
Infigratinib	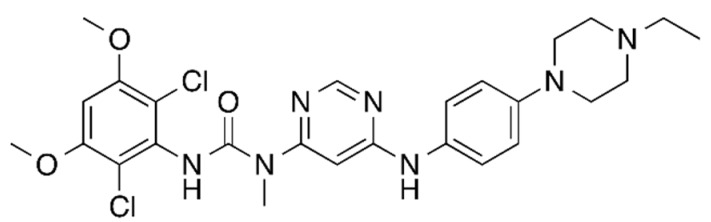	~1.1 nM [92]	~75% [93]
Lenvatinib	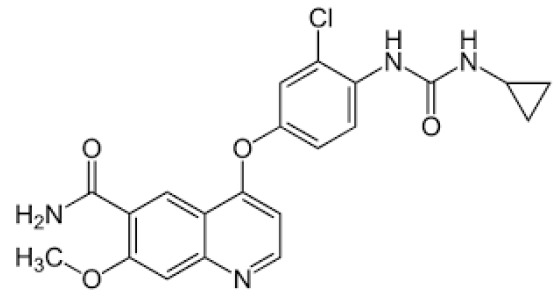	~46 nM [94]	85% [89]
Nintedanib	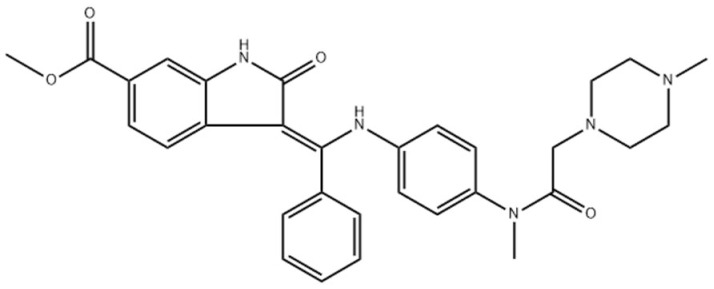	~69 nM	5%
Palifermin	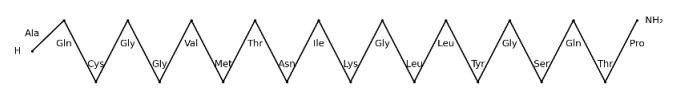	Not reported	Not reported
Pemigatinib	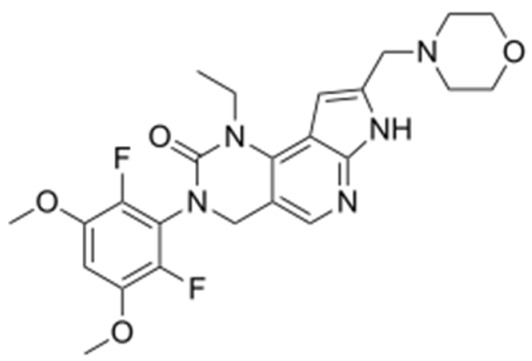	~0.4 nM	Not reported
Ponatinib	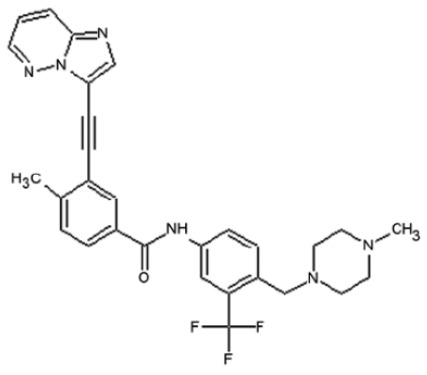	~2.2 nM [95]	54% [96]
Pralsetinib	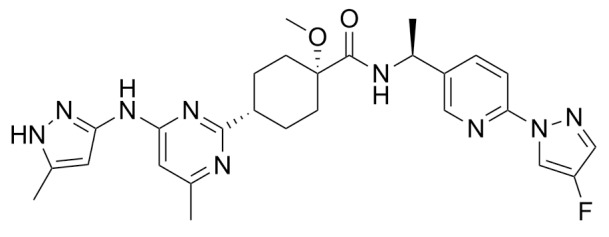	144–160 nM [97]	~70%
Regorafenib	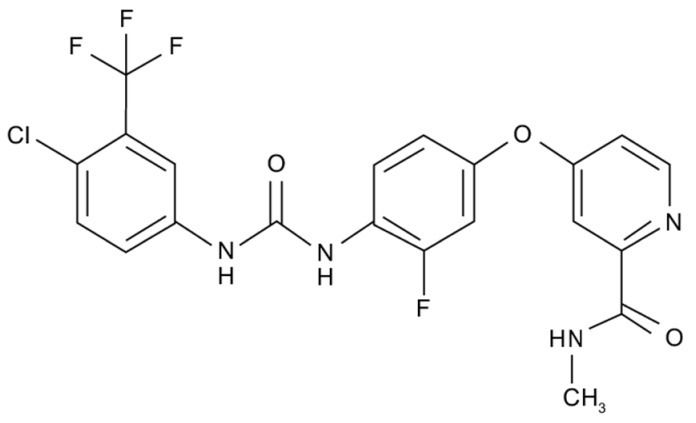	~202 nM [98]	69–83%
Romiplostim	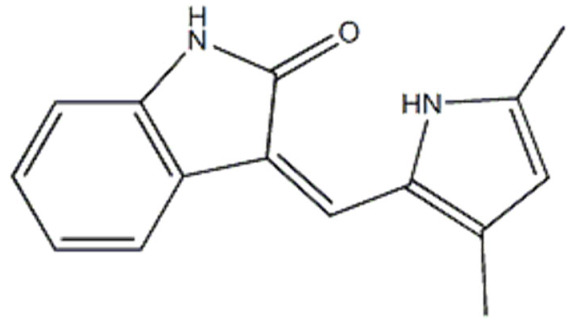	Not reported	~0%
Selpercatinib	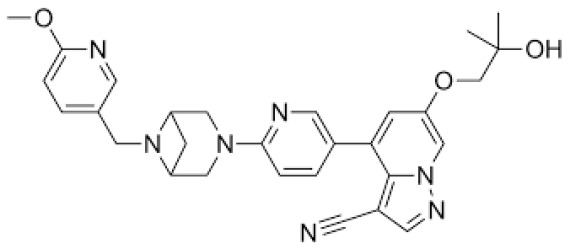	Not reported	73% [99]

**Corrected version of Table 2:**

**Table 2:** Chemical structures bioavailability of the compounds and IC_50_ of them with Fibroblast Growth Receptor 1.

Compound	Chemical Structures	IC_50_ with FGFR1	Bioavailability per os
Fostamatinib	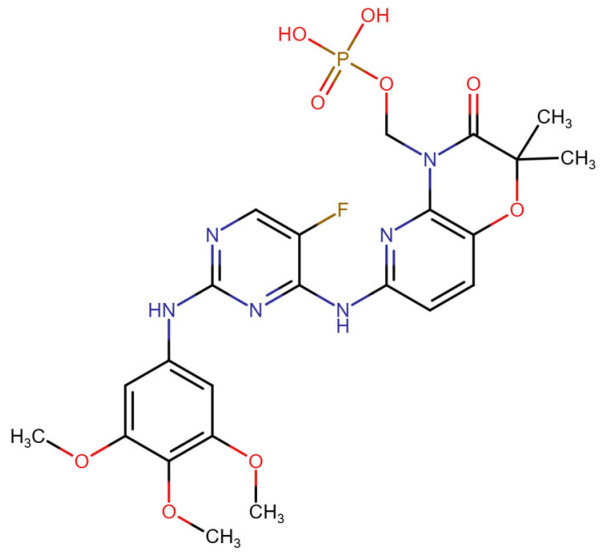	0.4 [87]	55% [88]
Erdafitinib	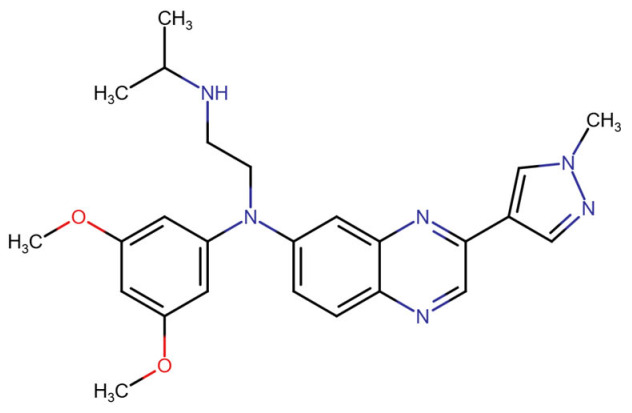	~2 nM [89]	60%
Futibatinib	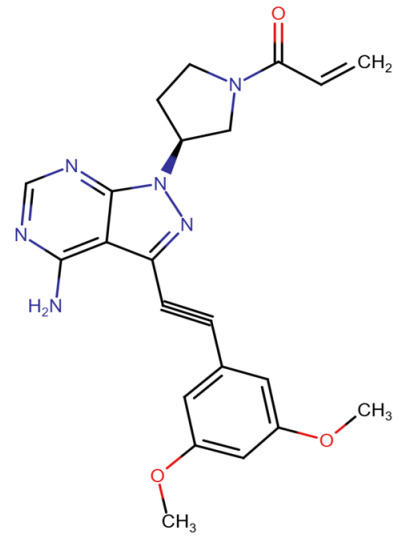	~1.4 nM [90]	79.8% [91]
Heparin	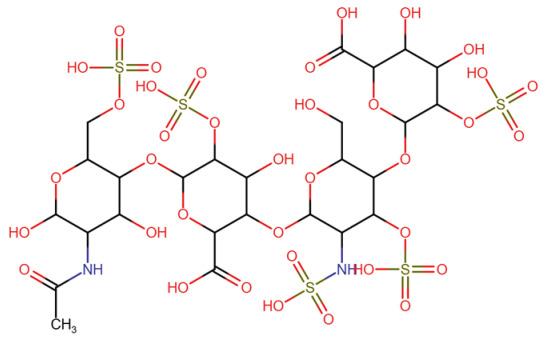	~63 nM [90]	Not reported
Infigratinib	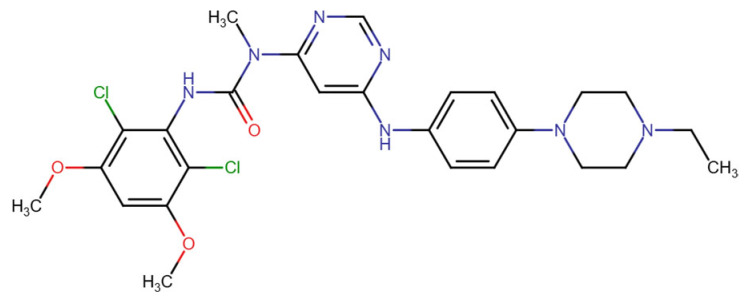	~1.1 nM [92]	~75% [93]
Lenvatinib	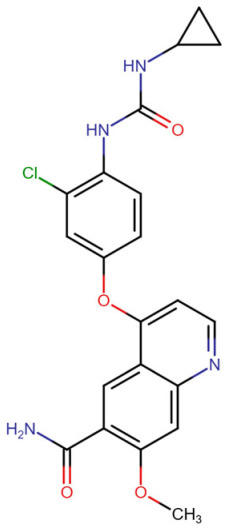	~46 nM [94]	85% [89]
Nintedanib	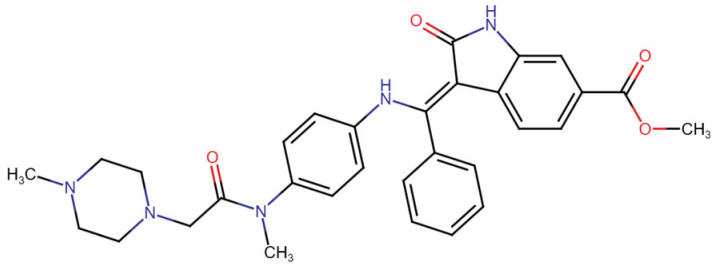	~69 nM	5%
Palifermin	C_721_H_1142_S_9_	Not reported	Not reported
Pemigatinib	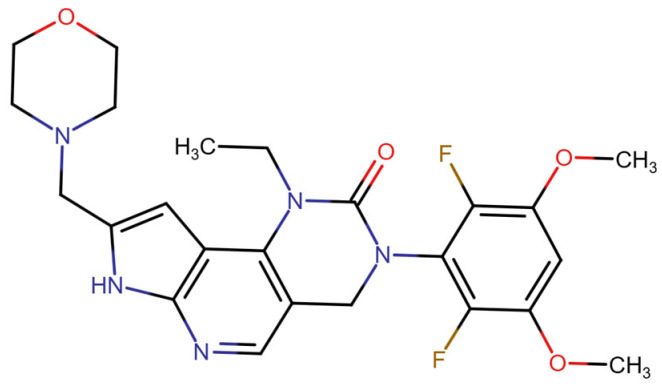	~0.4 nM	Not reported
Ponatinib	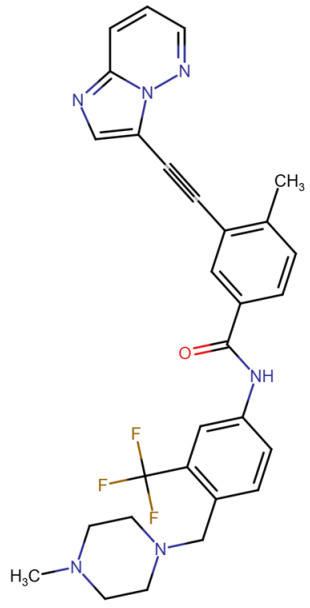	~2.2 nM [95]	54% [96]
Pralsetinib	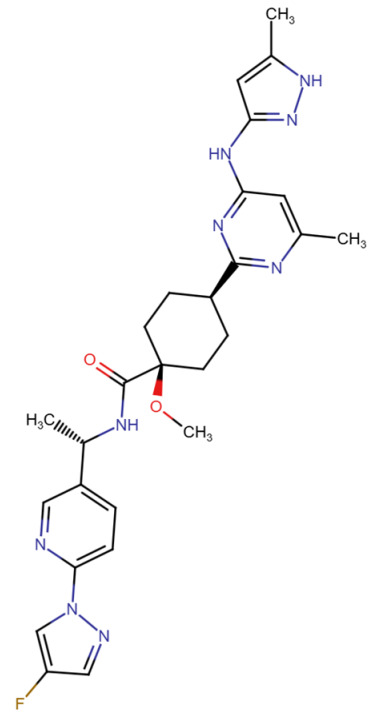	144–160 nM [97]	~70%
Regorafenib	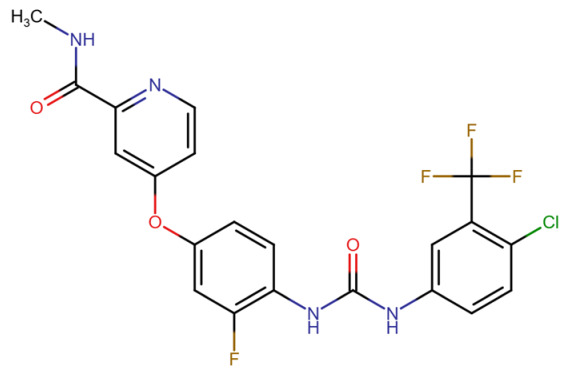	~202 nM [98]	69–83%
Romiplostim	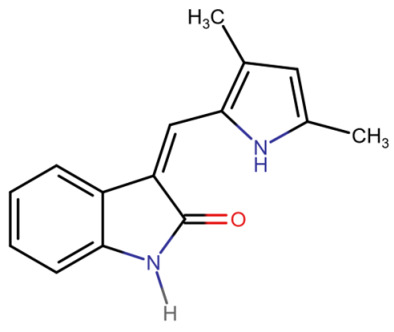	Not reported	~0%
Selpercatinib	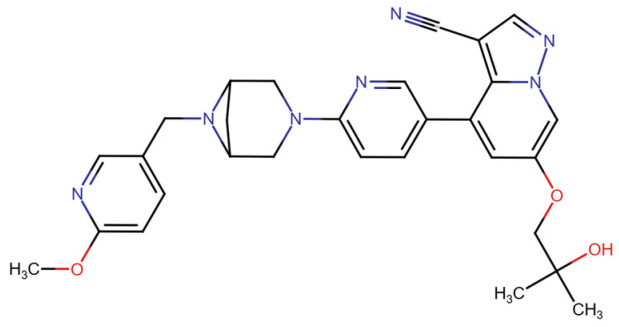	Not reported	73% [99]
